# Computational Discovery of Plant-Derived Peptides with Therapeutic Potential: A Scoping Review

**DOI:** 10.3390/ijms27219639

**Published:** 2026-10-28

**Authors:** Siphumelele Maphumulo, Nomakhosi Mpofana, Elizabeth Ojewole

**Affiliations:** 1 Department of Somatology, Durban University of Technology, Durban 4000, South Africa; siphumelelem1@dut.ac.za; 2 Department of Public Health, E-Health Sub-discipline, School of Medicine, College of Health Sciences, University of KwaZulu-Natal, 719 Umbilo Road, Durban 4001, South Africa; siphumelelem1@dut.ac.za; 3 Discipline of Pharmaceutical Sciences, School of Health Sciences, University of KwaZulu-Natal, Durban 4000, South Africa; ojewolee@ukzn.ac.za

**Keywords:** plant-derived peptides, antimicrobial peptides, bioactive peptides, machine learning, protein language models, molecular docking, scoping review, PRISMA-ScR

## Abstract

Computational methods are increasingly applied to the discovery of plant-derived peptides, but the extent, composition and reporting practice of this literature have not been mapped. Following PRISMA-ScR and Joanna Briggs Institute guidance, Scopus, Web of Science, ScienceDirect and PubMed were searched for records published between 2021 and 2026. Of 5062 unique records, 593 were screened at title and abstract, 88 full texts were assessed, 80 sources met all five eligibility criteria and 79 were charted. Overall, 49 (62.0%) were primary studies, 12 (15.2%) described a tool or database and 18 (22.8%) were narrative reviews. Antimicrobial peptides were the commonest class (33, 41.8%), followed by antioxidant (21, 26.6%) and ACE-inhibitory peptides (18, 22.8%). Only 11 sources (13.9%) trained and evaluated a model of their own, whereas 23 (29.1%) combined prediction with molecular docking. Of 61 empirical sources, 14 (23.0%) reported at least one quantitative prediction-task performance figure and 13 (21.3%) benchmarked a named comparator; the metrics used were not mutually comparable. Moreover, 18 of 62 applicable sources (29.0%) confirmed their own prediction experimentally; 70 (88.6%) named a plant source, most often *Arabidopsis thaliana*; and 8 (10.1%) linked their material to documented traditional medicine. No source applied machine learning to African plant peptides, an absence within the literature sampled rather than proof that none exists.

## 1. Introduction

Plants produce a structurally diverse repertoire of peptides, many of which have measurable biological activity. Two research traditions have converged on this repertoire. The first characterises the structural families of plant innate immunity: antimicrobial peptides, defensins, thionins, snakins, hevein-like peptides, lipid transfer proteins and cyclotides [1,2,3,4]. The second characterises bioactive sequences released by enzymatic hydrolysis of seed, leaf and by-product proteins, largely within food and nutrition science [5,6,7,8]. Peptides from both traditions inhibit bacterial and fungal growth [9,10,11], scavenge free radicals [12,13,14], inhibit angiotensin-converting enzyme [15,16], inhibit dipeptidyl peptidase IV and lower glucose [17,18], and modulate immune and inflammatory signalling [19,20]. Interest has intensified as antimicrobial resistance has eroded established anti-infective classes [3,4,21] and as demand has grown for functional food ingredients with demonstrable mechanisms of action [5,7,19].

Identifying these peptides experimentally is slow, and the candidate space is very large. Extraction, fractionation, purification, sequencing and bioassay of a single candidate can occupy months, while a single plant proteome yields tens of thousands of putative sequences once enzymatic cleavage is simulated across several proteases [6,22], and more still when small open reading frames, microRNA-encoded peptides and other non-canonical coding regions are counted [23,24,25,26]. Exhaustive experimental screening is therefore not merely expensive but infeasible, and candidates must be prioritised computationally before any peptide is synthesised.

Computational methods supply that prioritisation at several points in the discovery pipeline. Sequence-based classifiers assign a probability that a peptide belongs to a bioactivity class, and predictors have been trained specifically on plant material rather than on the taxonomically mixed datasets that dominate general antimicrobial peptide prediction [25,27,28,29]. Protein language models, pretrained on large unlabelled sequence corpora and fine-tuned on smaller labelled plant datasets, have more recently been applied to the same task [24,29,30]. Molecular docking estimates whether a prioritised sequence occupies a target binding site and with what energetic favourability [31,32,33]; molecular dynamics simulation tests whether the resulting complex remains stable [34,35]; and genome, transcriptome and peptidome mining identifies candidate loci and endogenous sequences [36,37,38,39,40,41]. These approaches are complementary rather than competing, and most published pipelines combine several of them.

As this literature has grown, so has its vocabulary. Studies and reviews alike describe themselves as artificial-intelligence-driven, machine-learning-guided or deep-learning-based [14,19,42,43,44], and such descriptions are frequently repeated in the secondary literature without scrutiny. Four things remain unestablished: how much of this work actually trains and evaluates a predictive model; how comparably its performance is reported; how often a computational prediction is confirmed experimentally by the study that generated it; and which plants the field draws upon. These concern the extent, composition and internal consistency of an evidence base rather than the efficacy of any single method, which is what a scoping review is designed to establish [45,46,47].

This scoping review maps computational approaches to the discovery of plant-derived peptides. For every included source it charts the peptide class or classes addressed, the computational method applied, the reported performance and the data split on which it was measured, whether the source experimentally confirmed its own prediction, the botanical provenance of the peptide material, and any link between that material and a documented traditional-medicine system.

## 2. Materials and Methods

### 2.1. Study Design

This scoping review adopted the five-stage methodological framework of Arksey and O’Malley [45], as refined by Levac and colleagues [46] and further updated by Peters and colleagues [47], and is reported in accordance with the PRISMA-ScR guideline [48]. Data extraction, analysis and presentation followed the recommendations of Pollock and colleagues for scoping reviews [49]. A completed PRISMA-ScR checklist is provided as Supplementary File S2.

A scoping design was selected in preference to a systematic review for two reasons. The object of enquiry is the extent, range and reporting practice of a heterogeneous methodological literature rather than the effect of a defined intervention; and the absence of a shared outcome metric across this literature precludes quantitative synthesis, so no pooled estimate could be generated from it.

### 2.2. Research Question

The review question was framed using the Population–Concept–Context framework recommended for scoping reviews [47] and is set out in Table 1: what computational approaches have been applied to the identification, prediction, classification or design of plant-derived peptides, and what is known about the performance and the experimental validation of these approaches? Two subsidiary questions followed from the charting form: from which plants does the field draw its material, and to what extent is that material linked to documented traditional-medicine knowledge? Geographic distribution was charted from the botanical origin of the peptide material rather than from the institutional affiliation of the authors, since affiliation records where research is conducted and not which flora it interrogates, and the two diverge systematically in this literature.

Two features of this framework are deviations from the protocol and are recorded as such. The Population element requires that the plant be the source of the peptide, which excludes molecular pharming, since a recombinant human or microbial peptide expressed in a plant chassis is not plant-derived. The Concept element admits any in silico approach rather than privileging machine learning, which would otherwise embed in the eligibility criteria the assumption the review set out to test; method type is therefore charted as an attribute and reported in Section 3.2.2.

### 2.3. Eligibility Criteria

Eligibility criteria were derived directly from the framework in Table 1 and are summarised in Table 2. A source was included only where all five criteria were satisfied; failure against any single criterion was sufficient for exclusion, and the criterion that determined each exclusion was recorded.

Two points of interpretation governed the application of these criteria. The computational-component criterion is deliberately asymmetric between primary studies and reviews: a narrative review that discusses computational approaches substantively is eligible, but it is charted as applying no method, since discussion is not application. The date restriction was applied prospectively at the point of searching rather than post hoc, which the exports confirm: of the 4886 records exported from Scopus, Web of Science and ScienceDirect, none carries a publication year before 2021.

### 2.4. Information Sources

Structured searches were conducted in four bibliographic databases, namely Scopus, Web of Science, ScienceDirect and PubMed, selected to provide complementary coverage of the biomedical, pharmaceutical, computational, food science and plant science literatures in which this field is dispersed. Scopus, Web of Science and ScienceDirect were searched on 17 August 2026, returning 3963, 540 and 383 records, respectively. PubMed was searched on 17 August 2026 and, following an amendment to the search string, again on 12 October 2026. The amendment corrected two faults in the original PubMed search: truncation operators placed inside quoted phrases, which PubMed does not expand, and a rolling five-year date filter in place of the fixed range from 2021 to 2026 applied to the other three databases. The original string returned 30 records and the amended string 431. The two sets are not nested, with 9 of the original 30 records being absent from the amended set, so both runs are reported and their union of 440 unique records is carried forward. Both strings, the fields searched, the date of each run and the yield of each are given in Supplementary File S1.

The four databases together yielded 5326 records. Records were exported in BibTeX format from Scopus and in RIS format from Web of Science and ScienceDirect; PubMed records were exported in abstract format. All export files have been retained and are the source of every count reported in Section 3.1.

### 2.5. Search Strategy

The search strategy was developed around the three concepts in Table 1. Search terms were organised into blocks covering, respectively, computational and in silico approaches; peptides and peptide families; and plants, plant parts and natural products. Terms within each block were combined with the Boolean operator OR and the blocks were combined with AND. Truncation was used to capture morphological variants. Searches were restricted to title, abstract and author keyword fields, and to the publication years 2021 to 2026. No language limit was applied at the point of searching; language was assessed as an eligibility criterion during screening. Complete database-specific strings, including both PubMed strings, are reported verbatim in Supplementary File S1 so that every search can be re-executed independently.

### 2.6. Study Selection

Records retrieved from the four databases were combined and duplicates removed using digital object identifiers (DOIs) and normalised titles. This process removed 264 duplicate records, leaving 5062 unique records for further assessment.

An initial keyword filter was applied to identify records for title and abstract assessment; the filter did not itself determine study eligibility. Records were retained where the title, abstract or author keywords contained at least one peptide-related term and one plant-related term. The filtering process was case-insensitive and included relevant variations of the search terms. The complete keyword lists are provided in Supplementary File S3. The filter retained 593 records for title and abstract screening. To assess its performance, the filter was tested against 24 previously identified eligible studies, all of which were retained.

**Figure 1:**
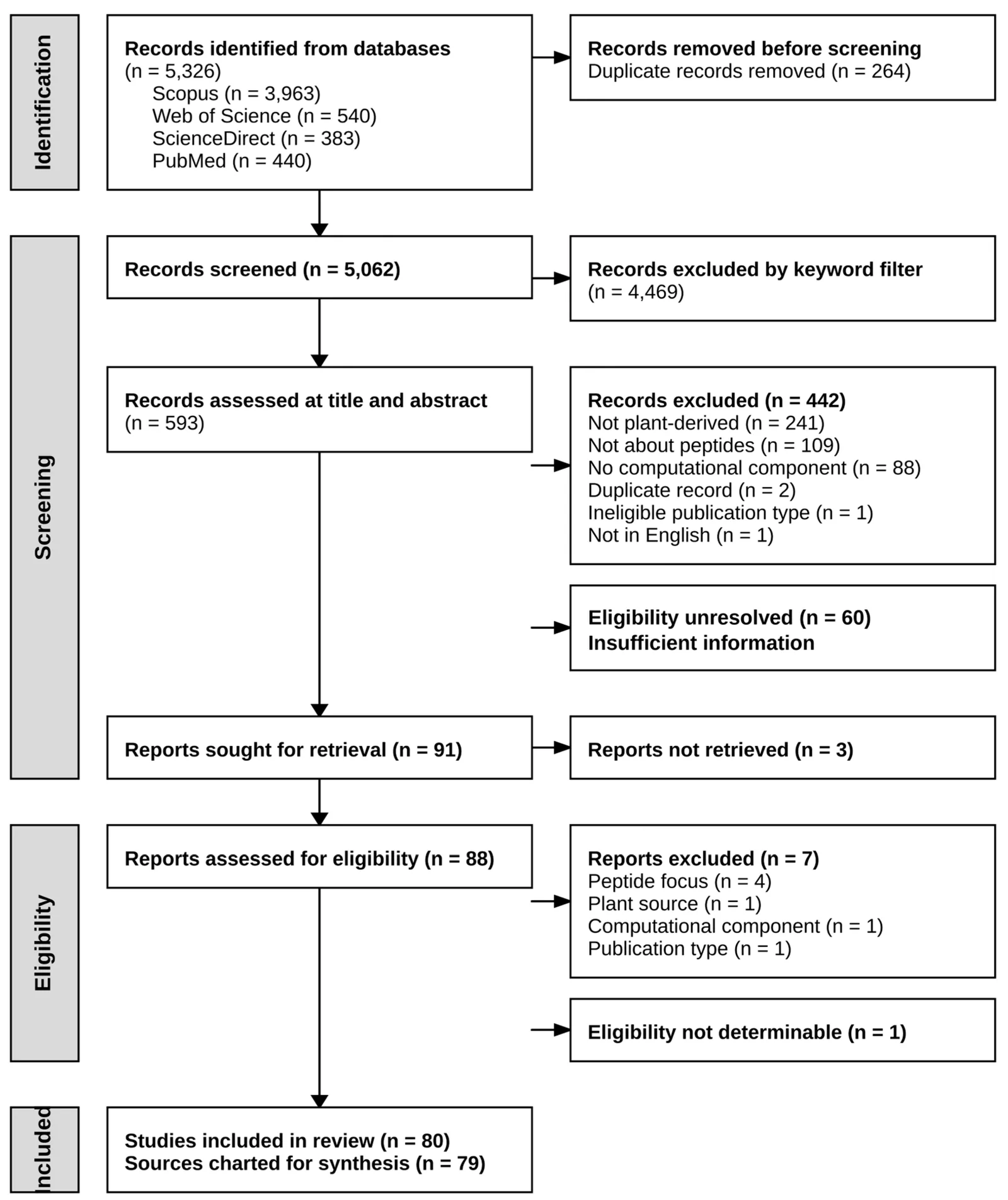
PRISMA 2020 flow diagram of the selection process.

The 593 records were screened against the five eligibility criteria outlined in Table 2. Of these, 442 were excluded for not meeting the inclusion criteria, 60 remained unresolved because the available information was insufficient to establish eligibility, and 91 progressed to full-text retrieval. The 60 unresolved records were not assessed as ineligible and were not advanced to full-text assessment; they are reported separately in Figure 1. Screening decisions and reasons for exclusion are documented in Supplementary File S3.

Full texts were sought for the 91 records that met the eligibility criteria during title and abstract screening. Of these, 88 reports were retrieved and assessed against the same five criteria, while 3 could not be retrieved because of restricted access. Following full-text assessment, 80 sources met the inclusion criteria, 7 were excluded and 1 remained unresolved because its eligibility could not be established from the available information. The screening and eligibility assessment outcomes, including supporting evidence and reasons for exclusion, are documented in Supplementary File S4. The study selection process is illustrated in the PRISMA 2020 flow diagram (Figure 1).

#### Reviewer Involvement and Screening Verification

S.M. and N.M. participated in the title, abstract and full-text screening process, applying the predefined eligibility criteria presented in Table 2. E.O. contributed to the verification of screening decisions and their consistency with the eligibility criteria. Screening outcomes were documented with reasons for exclusion and supporting evidence from the available records and full texts. An AI-assisted quality-assurance pass was subsequently used to examine available screening records and full-text evidence against the eligibility criteria and to support evidence-linked charting. This pass was not treated as an additional independent human review.

The retained screening records did not establish whether assessments were performed independently or how disagreements were resolved. Individual reviewer-level decisions were also unavailable for comparison; therefore, inter-reviewer agreement could not be calculated. These limitations are acknowledged in Section 4.6.

### 2.7. Data Charting and Extraction

A standardised charting form was applied to the 79 sources for which charting could be completed and captured the following fields: study type; peptide class or classes addressed; plant provenance; every computational tool, algorithm, model and database actually applied; the method category assigned on the basis of that list; the dataset used and its composition and size; the validation design; every reported performance metric at the value given by its source and labelled with the data split on which it was measured; the comparator tools, if any, benchmarked against; whether the source experimentally validated a prediction of its own; whether it linked its material to a documented traditional-medicine system; the principal finding; and any caveat affecting interpretation. The form was piloted on ten sources and refined once before full application. Where a field was not addressed by a source, “not stated” was recorded; no value was inferred from context.

Four operational definitions governed charting and determine the counts reported in Section 3.

First, the computational method was charted as applied rather than as discussed. A narrative review that surveys machine learning at length but trains no model is recorded as applying no method; a primary study that runs a published prediction server over a proteome is recorded as applying a previously published model, not as training one. Second, validation means experimental confirmation, by the source itself, of the biological activity of a specific peptide that the same source predicted or identified computationally. Computational cross-validation is not validation under this definition, since it tests a model rather than a peptide; nor is an assay performed on a bulk protein hydrolysate or an unresolved molecular-weight fraction rather than on the named sequences the computational step selected. Third, a source was recorded as linked to traditional medicine only where it states that the plant or peptide material has documented use within a named traditional-medicine system and cites a source for that use; description of a species as “medicinal” without a named system and a citation does not qualify. Fourth, in the species tally reported in Section 3.2.5, a species is counted for a source only where that source draws its own sequence data or plant material from it; species named as comparator genomes, background examples, structural analogues, expression hosts or supplementary coverage are excluded, as are species named by narrative reviews in discussing the work of others. Both the restricted and the unrestricted tallies are reported in Section 3.2.5 so that the effect of this rule is visible.

Charting was not performed in duplicate. Each source was charted once, from its full text, with AI-assisted support described in Section Reviewer Involvement and Screening Verification, and every charted value was required to carry a verbatim quotation from the full text as its evidential basis; values for which no supporting quotation could be identified were recorded as not stated. The quotations are reported alongside the charted values in Supplementary File S4, so that each value can be checked against its source. This is an evidence-linked single extraction with verification, and it is reported as such rather than as duplicate charting.

### 2.8. Data Synthesis and Analysis

Charted data were synthesised descriptively, which is the synthesis appropriate to a scoping review and the only synthesis the heterogeneity of this evidence permits. Frequencies and percentages are reported for study type, peptide class, method category, performance reporting, validation status, plant provenance and traditional-medicine linkage. Where a source addresses more than one peptide class it is counted under each, and where this produces a column total exceeding the number of sources it is stated explicitly. Percentages are calculated on the charted total of 79 unless a different denominator is named. Three denominators are used, each defined by the eligibility of a source for the analysis in question. The charted total of 79 is used for attributes that every source possesses, namely study type, peptide class, method as applied, plant provenance and traditional-medicine linkage. The 61 empirical sources, comprising the 49 primary studies and the 12 tool or database descriptions, are used for performance reporting and comparator benchmarking, because only sources that generate results of their own can report them. The 62 sources for which the validation question arises exclude the 17 narrative reviews that apply no computational method and therefore generate no candidate of their own; the single narrative review that did apply a prediction and docking pipeline is retained in that denominator. Each denominator is named at the point of use. Percentages are reported to one decimal place. No meta-analysis was attempted, no pooled effect estimate is offered, and no ranking of method families by performance is presented; Section 3.2.3 sets out in detail why the evidence does not support one.

### 2.9. Critical Appraisal of Included Evidence

Consistent with established scoping review methodology [47,48], formal critical appraisal of the methodological quality of included sources was not undertaken, since the purpose of a scoping review is to map the extent and nature of an evidence base rather than to grade the strength of its individual components. Omitting appraisal does not, however, require omitting the information on which appraisal would rest. In its place, this review charts reported performance, validation design, dataset composition and comparator benchmarking systematically and reports them at source values, which allows a reader to form an independent judgement of the evidential strength of any individual source from Section 3 and Supplementary File S4. The aggregate picture that emerges from those fields, namely that most empirical sources report no performance figure at all and that those which do are not mutually comparable, is reported as a finding in Section 3.2.3 rather than as an appraisal verdict.

### 2.10. Reporting and Protocol

This review follows the PRISMA-ScR guideline [48], and a completed 22-item checklist is provided as Supplementary File S2. No protocol was registered prospectively in a public registry prior to data extraction. This is a substantive limitation, since prospective registration is the principal safeguard against selective reporting and post hoc modification of eligibility criteria, and the eligibility criteria of this review were in fact modified during its conduct, as described in Section 2.2. The modifications and their consequences are reported in full in Section 4.6, and the complete decision record in Supplementary Files S3 and S4 is offered in partial mitigation, since it allows any reader to verify that the criteria as stated in Table 2 were applied uniformly to every record.

## 3. Results

### 3.1. Search and Screening Results

A total of 5326 records were identified across the four databases: 3963 from Scopus, 540 from Web of Science, 383 from ScienceDirect and 440 from PubMed, the last being the union of the two PubMed runs described in Section 2.4. Deduplication on digital object identifier and then on normalised title removed 264 records, leaving 5062 unique records. The documented keyword filter described in Section 2.6 retained 593 records for reading at title and abstract and excluded 4469, every one of which lacked either a peptide term or a plant term in its title, abstract or author keywords.

Of the 593 records assessed at title and abstract level, 442 were excluded, 60 could not be resolved using the available bibliographic information and 91 progressed to full-text retrieval. Exclusions at this stage were determined by plant source in 241 records, peptide focus in 109, absence of a computational component in 88, duplication not caught by the two deduplication passes in 2, ineligible publication type in 1 and language in 1. The unresolved records were predominantly exported without an abstract, leaving insufficient information in the title and author keywords to establish eligibility. They are displayed separately in Figure 1 because no eligibility verdict was reached.

Full texts were sought for all 91 records and obtained for 88. Three could not be retrieved, in each case because of a subscription paywall, and are reported as sought but not retrieved without an eligibility decision. Of the 88 reports assessed at full text, 80 satisfied all five criteria, and 8 did not proceed to inclusion: 7 were excluded, determined by peptide focus in 4 reports, plant source in 1, absence of a computational component in 1 and publication type in 1, and the eligibility of 1 further report could not be determined even from its full text. The complete selection pathway, with every count and every exclusion reason, is presented in Figure 1. All title and abstract decisions with their reasons are listed in Supplementary File S3, and all full-text decisions, with the evidence supporting each criterion-level verdict, in Supplementary File S4.

Of the 80 sources that satisfied all five eligibility criteria, 79 were charted. The available text for the eightieth source [50] was insufficient for the standardised data extraction, although the source was classified as eligible. No charted values were taken from it. Every count from Section 3.2 onwards is therefore stated out of 79 unless a narrower denominator is named. The resulting uncertainty is acknowledged in Section 4.6.

### 3.2. Characteristics of the Charted Evidence

Overall, 80 sources satisfied all five eligibility criteria, and 79 were charted. All were published between 2021 and 2026, consistent with the prospective date restriction. The distribution across years is markedly skewed towards the end of the window: 7 sources were published in 2021, 6 in 2022, 10 in 2023, 12 in 2024, 24 in 2025 and 20 in the part of 2026 covered by the search. Therefore, 44 of the 79 charted sources, or 55.7%, were published in 2025 or 2026. Because 2026 was only partially covered, these descriptive counts do not establish a publication growth rate. They nevertheless show that a substantial proportion of the charted sources appeared in the later part of the search window.

By study type, 49 sources (62.0%) were primary studies applying computational methods to a biological question, 12 (15.2%) were descriptions of a tool, predictor or database, and 18 (22.8%) were narrative reviews. The 49 primary studies and the 12 tool or database descriptions are treated jointly below as the empirical sources, a set of 61, since both generate results of their own and both can therefore be asked whether they report performance and whether they validate predictions. Counts are reported separately for the narrative reviews wherever that distinction is material.

#### 3.2.1. Peptide Classes

Antimicrobial peptides are the most frequently addressed class, appearing in 33 of the 79 charted sources (41.8%), where the class is taken to include sources addressing antimicrobial, antibacterial or bacteriocin-type peptides [1,3,4,11,27,28,29,31,32]. Antioxidant peptides follow in 21 sources (26.6%) [12,13,14,51,52,53,54] and angiotensin-converting-enzyme-inhibitory or antihypertensive peptides in 18 (22.8%) [15,16,18,55]. A further 18 sources (22.8%) address bioactive peptides without specifying a functional activity class [56,57,58,59].

Among the more specific classes, defensins are addressed in 12 sources (15.2%) [2,33,36,37], peptides with antidiabetic, dipeptidyl-peptidase-IV-inhibitory or other glucose-lowering activity in 11 (13.9%) [17,22,60,61,62] and anticancer peptides in 9 (11.4%) [51,63,64,65]. Antifungal peptides [25,27], antiviral peptides [66,67], immunomodulatory peptides [19,20] and cyclotides [4,68,69,70] are each addressed in eight sources (10.1%). Anti-inflammatory peptides [8,71], small secreted or small signalling peptides [2,26,30,72] and snakin or GASA-domain peptides [33,38,63] are each addressed in seven (8.9%), lipid transfer proteins in five (6.3%) [3,37], and peptides encoded by short open reading frames, microRNAs or other non-canonical coding regions in four (5.1%) [23,24,25,26], as are thionins [1,3] and neuroprotective peptides [35,73]. Cell-penetrating peptides are addressed in two (2.5%) [63,65]. Four sources address classes falling outside this scheme entirely: ribosomally synthesised and post-translationally modified peptides, specifically cyclopeptide alkaloids and related burpitides, in two [39,41]; cosmetic peptides in one [44]; and anti-fatigue peptides in one [74]. Because most sources address more than one class, these figures sum to considerably more than 79; the full distribution is given in Table 3 and the per-source assignment in Supplementary File S4.

Antimicrobial peptides, while still the single most frequently addressed class, do not dominate it. Antioxidant and ACE-inhibitory peptides together account for 39 class assignments, a figure comparable to the 33 for antimicrobial peptides, and both classes belong overwhelmingly to the food science literature on protein hydrolysates rather than to anti-infective research. Computational peptide discovery in plants is thus being pursued as vigorously in food and nutrition science as in pharmacology. The unspecified category is also substantial: 18 sources, nearly a quarter of the evidence base, address bioactive peptides without committing to a functional class. In some cases, this reflects a genuinely exploratory design, in which a hydrolysate is profiled against several activity predictors simultaneously; in others, it reflects imprecise reporting. The charted records distinguish the two, but the frequency of the category is itself an indicator of how loosely activity class is specified in this literature.

#### 3.2.2. Computational Methods as Applied

Classifying each source by the method it actually applied, rather than by the methods it discusses, produces the distribution in Table 4. The single largest category is bioinformatics prediction combined with molecular docking, applied by 23 sources (29.1%), a pipeline that typically runs one or more published activity predictors over a set of hydrolysate-derived or proteome-derived sequences and then docks the highest-scoring candidates against a target protein [9,14,16,20,53,71,75]. The second largest category, at 17 sources (21.5%), applied no computational method at all: these are the narrative reviews, which discuss computational approaches, sometimes at considerable length, without undertaking any [1,3,4,5,7,21,26,42,43,76]. Nine sources (11.4%) applied previously published prediction models within a pipeline of their own without training or evaluating any model [52,63,65,67,77,78,79]. Seven (8.9%) applied conventional bioinformatics and gene-family analysis, typically genome-wide identification and phylogenetic characterisation of a peptide family [36,37,38,41,57,80], and a further seven (8.9%) applied molecular docking and simulation alone, without a preceding prediction step [31,33,34,35].

Eleven sources (13.9%) trained and evaluated a machine learning model of their own. Within this group, three degrees of model development can be distinguished, and the distinction matters for what each source contributes. Two fine-tuned the weights of a pretrained model, one by prompt-guided staged fine-tuning of a protein language model [29] and one by transfer learning on a pretrained recurrent network [81]. Four used a pretrained protein language model as a fixed feature extractor and trained a new downstream classifier on its embeddings [13,24,30,82]. Five trained a model from scratch with no pretrained component, comprising two deep learning architectures [25,72], two classical ensembles [15,28] and one single classical classifier [27]. Two further sources applied deep learning to structure prediction rather than to discovery or classification [44,68], two described a database or curated resource without a predictive model [58,83] and one applied a rule-based in silico simulation [64].

Although this field describes itself, in its titles, abstracts and reviews, as machine-learning-driven and artificial-intelligence-guided, machine learning that is trained and evaluated by the source reporting it accounts for 11 of 79 charted sources, or 18.0% of the 61 empirical sources. The great majority of the literature applies existing predictors, performs molecular docking, or conducts conventional bioinformatic characterisation. Applying a well-validated predictor to a new plant proteome and characterising a gene family are productive research strategies in their own right. The difficulty is that the published framing does not distinguish these activities from model development, and the distinction is invisible to a reader unless method is charted as applied rather than as described. Two consequences follow. A reader cannot infer from the language of an abstract whether a study built a predictor or used one; and the apparent methodological maturity of the field is, in aggregate, overstated by its own vocabulary.

#### 3.2.3. Reported Performance

Of the 61 empirical sources, 14 (23.0%) report at least one quantitative performance figure for a prediction task, 2 (3.3%) report structure-prediction confidence scores only, and 45 (73.8%) report none. The distribution is summarised in Table 5.

Disaggregating by what each source set out to do changes the picture materially, and it is the more informative comparison. Of the 11 sources that trained and evaluated a model of their own, 10 report at least one metric; the eleventh describes an 80:20 training and test split and a fusion of 10 algorithms but reports no numerical result for any of them [13]. The aggregate totals in Table 5 leave 4 of the 50 other empirical sources reporting a quantitative prediction-task performance figure; two additional sources report structure-confidence scores only. Reporting practice among model-development studies is therefore substantially complete, and the shortfall lies almost entirely with sources applying published predictors. This is a weaker criticism of the field than an undifferentiated figure implies, since a study applying a validated tool is not obliged to re-evaluate it; but it remains consequential, because without any evaluation a reader cannot tell whether the tool was appropriate to the material it was applied to.

The metrics reported are not comparable with one another. Across the 16 reporting sources, the quantities reported include accuracy, balanced accuracy, sensitivity, specificity, precision, recall, F1 score, the Matthews correlation coefficient, the area under the receiver operating characteristic curve, the area under the precision–recall curve, the coefficient of determination, root mean squared error, mean absolute error and a Pearson correlation coefficient. These are not simply different choices from a common menu. Some sources frame prediction as a classification problem and report accuracy and the Matthews correlation coefficient [27,28,29]; others frame the same underlying task as regression and report the coefficient of determination and root mean squared error [81]. One source reports balanced accuracy, which is the arithmetic mean of sensitivity and specificity and cannot be placed on the same scale as a plain accuracy computed on an imbalanced test set [25]. Two sources report structure-prediction confidence scores, which quantify a model’s confidence in a predicted fold and are not measures of predictive performance on a bioactivity task at all [44,68]. No single metric is reported by all 16 sources, and none is reported by even half of them.

Validation designs are no less heterogeneous. They range from a single train–test split with no cross-validation, through five-fold and ten-fold cross-validation, to ten-fold cross-validation combined with multiple independent test sets and twenty repeat runs [28,29]. Several sources report a metric without stating the data split on which it was measured, which makes the figure uninterpretable even in isolation, since a cross-validated accuracy and a held-out test accuracy are different quantities. Benchmarking against existing tools is the exception rather than the rule: 13 of the 61 empirical sources (21.3%) benchmark against at least one named computational comparator, and where comparators are reported they are generally reported well [25,27,28,29], but the remaining 48 name none. Even among those that do, comparison against a differently constructed dataset is not a like-for-like comparison, since a tool evaluated on another tool’s positive and negative sets inherits that construction’s redundancy thresholds and negative-sampling assumptions.

The consequence is unambiguous. This review cannot rank method families by performance, and neither can any other reader of this literature, because a ranking requires a common metric measured on a common benchmark and neither exists. This is not a finding that the methods perform poorly, which would require the evidence that is missing, but that their relative performance is not determinable from the published record. Section 4.3 sets out the minimal reporting convention that would remedy this.

#### 3.2.4. Validation of Computational Predictions

Applying the definition in Section 2.7, 18 of the 62 sources for which the question arises (29.0%) experimentally confirmed the biological activity of a specific peptide that the same source had predicted or identified computationally; 44 (71.0%) did not. For the remaining 17 charted sources, the question does not arise, with these being narrative reviews that apply no computational method and therefore generate no candidate of their own. The one narrative review that did apply a prediction and docking pipeline is retained in the denominator of 62.

The 18 validated cases follow a consistent and methodologically coherent pattern: computational screening narrows a large candidate set to a small number of named sequences, those specific sequences are chemically synthesised, and their activity is then measured in a relevant assay [14,15,16,61,75]. Several go substantially further, carrying a named peptide through enzyme-kinetic characterisation, cell-based assays or an animal model [25,62,84]. Validated cases are concentrated in the food science literature on antioxidant, antihypertensive and antidiabetic peptides, where synthesising and assaying predicted sequences is routine practice. Where a field has an established convention of experimental follow-through, the validation rate reflects it.

One pattern among the 44 unvalidated sources is easily mistaken for validation and is sometimes described as such by the sources themselves. A substantial number perform in vitro assays, but on a bulk protein hydrolysate or an unresolved molecular-weight fraction rather than on the specific sequences that the computational step selected. Such work establishes that the mixture possesses the activity in question; it does not establish that the predicted peptides are responsible for it, since a hydrolysate contains many sequences and the activity may reside in any of them, in several jointly, or in a component that was never predicted at all. Under the definition in Section 2.7 these sources are recorded as unvalidated, and the specific reason is recorded against each in Supplementary File S4. The distinction is between a tested prediction and an untested one.

#### 3.2.5. Plant Provenance

Overall, 70 of the 79 charted sources (88.6%) name the botanical origin of their peptide material; 9 (11.4%) do not, drawing instead on general peptide databases or on multi-species sequence sets whose constituent species are not enumerated [27,28,29,31]. Provenance is therefore stated in the great majority of sources.

The identity of the species named is, however, the more informative finding, and it depends on how the tally is constructed. Counting any mention of a species anywhere in the charted provenance field, across all 79 sources, *Arabidopsis thaliana* appears in 15 sources, followed by *Solanum lycopersicum*, *Medicago truncatula* and *Solanum tuberosum* in six each, *Oryza sativa* and *Vitis vinifera* in five each, and *Glycine max* in four. That tally is inflated, however, by narrative reviews listing species studied by others and by primary studies naming species only as comparator genomes or background examples. Restricting the tally by the rule stated in Section 2.7, so that a species is counted only where a source draws its own sequence data or plant material from it, and restricting the denominator to the 61 empirical sources, *Arabidopsis thaliana* remains the most frequent source species, in six sources [24,25,30,41,77,82], followed by *Medicago truncatula* [63,72,85] and *Solanum tuberosum* [33,63,77] in three each, and then by *Hordeum vulgare* [17,78], *Hibiscus syriacus* [39,63], *Pinellia ternata* [55,58], *Sesamum indicum* [15,63] and *Vitis vinifera* [33,77] in two each. Beyond these, the distribution has a long tail of single-source species, which is consistent with a field in which most primary studies characterise one plant.

Whichever tally is used, the profile is dominated by model organisms and by major agricultural crops. *Arabidopsis thaliana*, the most studied species in this literature on either count, is primarily a model organism rather than a species generally selected on the basis of medicinal use; its prominence may reflect the depth of its genomic, transcriptomic and annotation resources, and the consequent ease of assembling training and test data, rather than any pharmacological interest in the plant. Similar considerations may apply to tomato, potato, rice, maize, soybean, barley and grapevine, although the charted data do not establish why individual research groups selected these species. The evidence shows frequent use of genome-rich model organisms and crops, rather than demonstrating a consistent selection process based on documented therapeutic use. The nine sources that name no provenance at all reinforce the same point from the opposite direction, since a predictor trained on a taxonomically unspecified sequence set cannot be interrogated for the botanical applicability of its predictions.

#### 3.2.6. Linkage to Traditional Medicine

Overall, 8 of the 79 charted sources (10.1%) link their peptide material to a documented traditional-medicine system under the rule stated in Section 2.7, which requires the source both to name a system and to cite support for the use [16,43,53,55,58,68,73,83]. The systems named are Traditional Chinese Medicine, Ayurveda, Unani and the Indian Siddha system. Seventy-one sources (89.9%) make no such link.

A number of further sources describe a species as medicinal, or as traditionally used, without naming a system or citing any support for the claim. These are recorded as not linked, and the reason is recorded against each in Supplementary File S4. An unreferenced assertion that a plant is medicinal is not traceable to any body of knowledge a reader could consult, and counting such statements would inflate this figure substantially.

Computational discovery of plant-derived peptides is therefore, at present, only weakly connected to documented ethnomedical knowledge, with approximately one source in ten making the connection explicit. This is a missed opportunity rather than merely an absence. Traditional-medicine systems encode centuries of systematic observation about which plant materials exert biological effects, and such observation is precisely the kind of prior information that a candidate-selection step is designed to exploit. A pipeline that prioritises proteomes by documented ethnomedical use would be no more computationally expensive than one that prioritises by genome availability, and it would be interrogating a materially different hypothesis space.

#### 3.2.7. Geographic Scope of the Plant Material

Charted by the botanical origin of the peptide material rather than by author affiliation, no source within the scope of this search applied machine learning or deep learning to peptides from South African or other African plants. The species studied are concentrated in model organisms and in crops of global, East Asian or South Asian agricultural importance, as Section 3.2.5 sets out. A small number of sources name species of African distribution, but none combines African plant material with a trained or applied machine learning model.

This finding must be read against the design of the search, which is examined in Section 4.5. The search strategy contained no African-specific geographic, botanical or ethnomedical terms. No regional or continental database, such as African Journals Online or SciELO, was searched. No grey literature, theses, conference proceedings outside the four indexes or institutional repositories were consulted, and no preprint server was searched. Research published in regional journals that are not indexed in Scopus, Web of Science, ScienceDirect or PubMed could not have been retrieved by this search irrespective of its quality or relevance. The absence reported here is therefore an absence within the indexed international literature as sampled by this specific search, and it is not evidence that no such work exists.

## 4. Discussion

### 4.1. Summary of Main Findings

**Figure 2:**
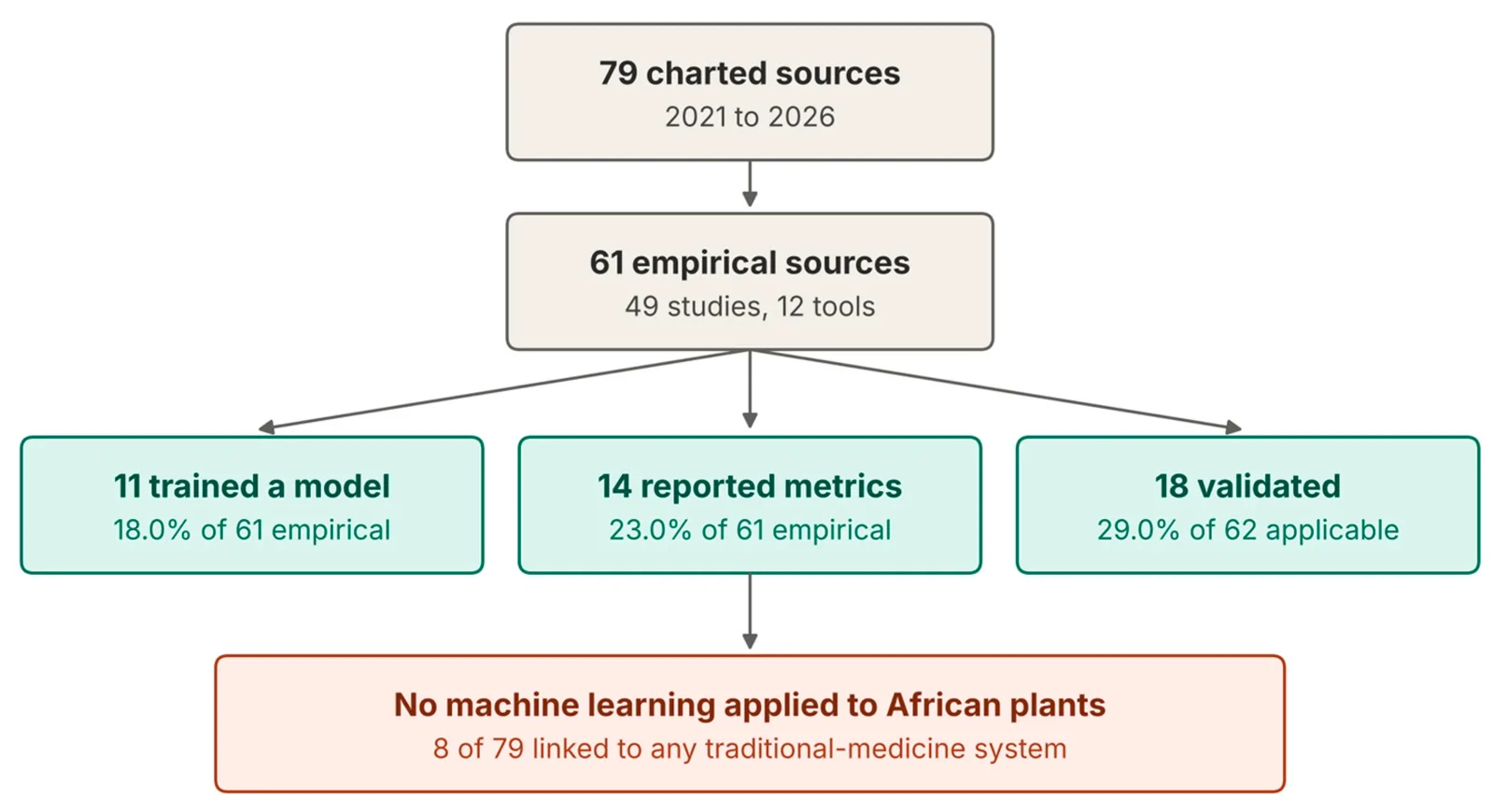
Attrition from the charted evidence base to methodological practice. Of the 79 sources charted, 61 reported results of their own; of these, 11 trained and evaluated a machine learning model, 14 reported a quantitative performance figure for a prediction task, and 18 of the 62 sources for which the question arises confirmed experimentally the activity of a peptide they had themselves predicted. Each proportion is expressed against its own denominator, and the groups overlap: four sources both trained a model and confirmed its prediction experimentally. The absence of machine learning applied to African plant material is an absence within the indexed international literature sampled by this search rather than evidence that no such work exists (Section 4.5).

This review charted 79 sources addressing computational approaches to the discovery of plant-derived peptides, identified through a documented search of four bibliographic databases. Five findings emerge, and they are interdependent rather than separate. Figure 2 presents the evidence in stages, starting with the studies included in the review. It then shows which studies generated original results, trained machine learning models, reported performance measures or experimentally validated predictions. Lastly, it highlights two key research gaps discussed in the following sections: the limited integration of ethnomedical knowledge and the underrepresentation of African medicinal plants.

First, the quantity of machine learning in this field is substantially smaller than its framing implies. Overall, 11 of 79 charted sources, or 18.0% of the 61 empirical sources, trained and evaluated a model of their own. The single largest method category, 23 sources, combined bioinformatics prediction with molecular docking; a further 9 applied models published by others without evaluating them, and 17 applied no computational method at all while discussing computational approaches at length. Those 17 are narrative reviews; the 18th review applied bioinformatics prediction with molecular docking and is charted under that category. This pattern becomes visible only when method is charted as applied rather than as described.

Second, performance reporting is not merely inconsistent but structurally incomparable. Overall, 14 of 61 empirical sources report any quantitative figure for a prediction task, and those that do report quantities drawn from different statistical frameworks: classification metrics from some, regression metrics from others, and structure-confidence scores from a third group that are not performance measures at all. Only 13 sources benchmark against a named computational comparator. No ranking of method families is supportable from this evidence, and none is offered here.

Third, experimental self-validation occurred in 18 of the 62 sources for which the question arises, which confirmed experimentally the activity of a peptide they had themselves predicted. These are concentrated in a specific sub-literature, as Section 4.4 discusses.

Fourth, botanical provenance is usually stated, in 70 of 79 sources, but the species named are predominantly model organisms and major crops. *Arabidopsis thaliana* is the single most frequently used source species on both the restricted and the unrestricted tally, despite having no medicinal use. This pattern may reflect the greater availability of genomic and annotation resources for model organisms and major crops, although the charted data record which species were used and not why they were chosen.

Fifth, linkage to documented traditional-medicine knowledge is rare, at 8 of 79 sources. Taken with the fourth finding, this indicates that a large body of prior observation about plant bioactivity is little used as a source of candidate material in this literature.

### 4.2. Antimicrobial Peptides and the Methods Used to Identify Them

Antimicrobial peptides remain the most frequently investigated class, in 33 of 79 sources, which is unsurprising given the clinical urgency of antimicrobial resistance and the long-standing recognition of antimicrobial peptides as effectors of plant innate immunity [1,3,4]. What the charted evidence shows, however, is that the computational approaches applied to this class vary enormously in sophistication and, more importantly, in evidential strength.

At one end of that range are tools that train a classifier on curated plant-specific antimicrobial peptide datasets, evaluate it under cross-validation and on independent test sets, and report a full metric set against named comparator tools [27,28,29]. Such work is directly assessable: a reader can see what was trained, on what, and how well it performed relative to alternatives. At the other end are studies that run one or two published prediction servers over a proteome or a hydrolysate and report the resulting candidate counts, sometimes with docking energies attached, without any evaluation of the predictions themselves [9,11,31,32]. Such work may well be useful, and its candidates may well be real, but nothing in it bears on whether the predictions are reliable.

The difficulty is that both ends of this range are described in the literature in closely similar language. A reader encountering the phrase “machine-learning-guided identification of antimicrobial peptides” in a title or abstract cannot determine which of the two they are about to read. Section 3.2.2 separates them explicitly, and the separation matters for cumulative progress: a field cannot improve its predictors if the published record does not distinguish building a predictor from using one.

Set against the wider antimicrobial peptide literature, this profile is distinctive. Reviews of artificial-intelligence-driven antibiotic discovery describe a field in which model development, in silico screening and experimental confirmation are increasingly integrated into single pipelines, and in which generative and discriminative models are trained specifically for the task [86]. The plant-specific literature mapped here agrees with that account at its upper end: the dedicated plant antimicrobial peptide predictors train on curated plant data, benchmark against named comparators and report full metric sets [25,27,28,29]. It diverges from that account in aggregate, because such sources are a small minority of the whole.

The contrast is sharpest when the plant-specific literature is set beside the general antimicrobial peptide field from which its tools are largely borrowed. That field has been subjected to systematic comparative assessment, in which published predictors are re-evaluated on common datasets under a common protocol and their relative performance reported [87]; it maintains shared curated repositories that supply the positive data on which such comparisons rest [88], and it contains worked examples of the complete cycle, in which a model is trained, its candidates are synthesised, and their activity is confirmed in vitro and in animal models [89,90]. Each of these three elements is present in the plant-derived literature, but sparsely. Overall, 13 of the 61 empirical sources benchmark against a named computational comparator, and 2 sources evaluate a general predictor on plant data [25,29], but no independent comparative assessment of plant-specific predictors on a common dataset has been published. Curated repositories exist for general antimicrobial peptides [88] and for food-derived sequences [91], but no plant-specific resource of comparable standing underpins the predictors charted here. And 4 of the 11 sources that trained a model of their own also confirmed experimentally the activity of a peptide that model selected [13,15,25,81], so the complete cycle does occur, in roughly 1 source in 20 of the empirical total.

Two explanations for the divergence are consistent with the charted data. First, labelled plant-specific training data are scarce: the dedicated predictors draw on a small number of curated resources, and several sources use multi-species sets whose plant content is not enumerated [25,27,28,29]. Second, the question most of these studies ask is biological rather than methodological: they seek candidate peptides from a particular species, for which an established predictor is an appropriate instrument. The charted data do not record the disciplinary background of the investigators, so no inference is drawn from them.

The practical implication is that authors, reviewers and readers need method-as-applied reporting rather than method-as-described framing, and the gap this leaves is specific. Whether the general predictors that most of these studies apply transfer reliably to plant sequences has been tested only twice in this evidence base. Where it was tested directly, a general antifungal predictor evaluated on plant peptides returned an area under the curve of 0.78 and an accuracy of 74.96%, but a precision of 33.47% and a false-positive rate of 66.5% [25]. A single result cannot establish a general pattern, but it indicates that transfer should be quantified rather than assumed, and that aggregate accuracy may conceal a precision cost on plant data.

### 4.3. Performance Reporting and What Would Remedy It

Roughly three quarters of the empirical sources report no performance figure, and among those that do, no single metric is shared by even half. This is, on the evidence assembled here, the most consequential obstacle to progress in this field, because it means that the relative merits of competing approaches cannot be established from the published record, not with more reading and not with a more sophisticated synthesis. The obstacle is not one of insufficient research activity; Section 3.2 shows the literature expanding rapidly. It is an obstacle of incommensurability.

The remedy requires no new methods, only a reporting convention, and four items would be sufficient. First, dataset composition, giving the number of positive and negative sequences, the source of each, the redundancy threshold applied and the negative-sampling strategy used. Second, the validation design, stating explicitly whether an independent test set was held out, how it was constructed and how large it was, and distinguishing cross-validated figures from held-out figures. Third, the metric set, with each figure labelled by the data split on which it was measured, and with at least one metric robust to class imbalance such as the Matthews correlation coefficient or balanced accuracy reported alongside accuracy. Fourth, at least one named comparator tool evaluated on the same data by the authors themselves rather than quoted from the comparator’s own publication. Examples of reporting practices relevant to these recommendations are found in studies of dedicated plant peptide predictors [25,27,28,29]. Adopting it requires no new methodology for the reporting itself, although constructing an independent test set or evaluating a comparator on the same data may require additional data and computation.

These four items are not a novel prescription. They correspond closely to established reporting recommendations for supervised machine learning in biology, which call for explicit description of the data, the optimisation procedure, the model and its evaluation, including the independence of any test set and the choice of metrics appropriate to class balance [92], and to the parallel recommendations on reproducibility, which emphasise that a reported figure is interpretable only when the data split and the code that produced it are specified [93]. The present findings therefore do not identify a new problem so much as show that a recognised one is largely unaddressed in this particular literature: the sources that satisfy most of these recommendations are the dedicated plant peptide predictors [25,27,28,29], while the larger body of applied work satisfies few of them.

The heterogeneity of framing compounds the problem: a source that treats bioactivity prediction as regression reports the coefficient of determination and root mean squared error entirely appropriately for its own design, yet those values cannot be set beside the accuracy and Matthews correlation coefficient reported by a source that frames the same task as classification. Incommensurability of this kind is a structural feature of a field assembled from several disciplines, not a failure of any individual study.

A shared benchmark dataset for plant-derived peptide prediction would be a further and larger step, and the charted evidence shows why it is needed. Several sources construct their own positive and negative sets from the same underlying public databases, but with different redundancy thresholds, different length filters and different strategies for generating negatives, whether by random sampling from non-annotated proteome regions, by sequence shuffling or by selection from unrelated functional classes. These choices materially affect every metric computed downstream. The practical consequence is that two tools reporting the same accuracy on “plant antimicrobial peptides” may not be comparable at all, and a reader has no way to adjust for the difference. A community benchmark with fixed splits, published negatives and a stated evaluation protocol would convert a literature of incomparable claims into a cumulative one.

Such a benchmark is neither hypothetical nor unprecedented. Comparative assessments of general antimicrobial peptide predictors have already been performed on common datasets under a stated protocol, and they report that measured performance depends strongly on how the evaluation set is constructed [87]. The curated resources that make such work possible also exist for general antimicrobial peptides [88] and, for food-derived bioactive sequences, in databases that supply both the reference peptides and the in silico hydrolysis tools on which much of the literature charted here depends [91]. What is absent is a plant-specific benchmark of comparable standing. Constructing one would require no new methodology, only the agreement of a small number of groups that already hold the relevant data.

### 4.4. Validation and the Hydrolysate Problem

Overall, 18 of the 62 applicable sources confirmed experimentally the activity of a peptide they had themselves predicted. The validated cases are concentrated in the food science literature on protein hydrolysates, where synthesising a small number of candidate sequences and measuring their activity in a standard assay is established practice [14,15,16,61,62,75]. That concentration is instructive: experimental follow-through is not a function of computational sophistication but of disciplinary convention, and the convention in question is a transferable one.

One specific practice should be named, because it is easily mistaken for validation and is sometimes so described. A substantial number of sources perform in vitro assays on a bulk protein hydrolysate or an unresolved molecular-weight fraction rather than on the named sequences the computational step selected and then present the computational predictions as validated. They are not validated. The assay establishes that the mixture possesses the activity; it does not establish that the predicted peptides account for it. The activity may reside in a predicted peptide, in several of them jointly, in an unpredicted peptide, or in a non-peptide component of the fraction, and the experiment as performed cannot distinguish these possibilities. Synthesis of named candidates is not always feasible, and a hydrolysate-level assay is often an appropriate first step; where the specific sequences have not been tested, that should be stated rather than left for a reader to infer from the methods section.

The shape of this bottleneck differs from the one usually described for natural-product discovery at large. In that wider field, the limiting steps are classically isolation, structural elucidation and supply of sufficient material, and computational methods are advocated precisely because they reduce the number of compounds that must be carried through those steps [94]. In the literature mapped here, the computational step is widely adopted and frequently the less resource-intensive of the two, while the confirmatory step that would give it meaning is often omitted. This is a matter of degree rather than of kind: training a large model or running extended simulation is itself costly, and synthesis, purification and bioassay impose real financial and technical constraints. Within those limits, however, prediction has outrun confirmation rather than relieved it.

The omission is not always dictated by cost or feasibility. Published work on antimicrobial peptides from non-plant sources demonstrates the complete cycle at scale: candidate sequences generated by a trained model are synthesised, assayed against defined organisms, and in several cases carried through to animal infection models, with the proportion of predicted candidates that proved active reported explicitly [89,90]. Reporting that proportion is what converts a prediction pipeline into an instrument of known reliability, and it is precisely what the plant-derived literature does not yet provide.

What this leaves unknown is quantitative and consequential. The 18 validated sources establish that computationally selected plant peptides can be active; they do not establish how often predicted peptides prove active. Two distinct quantities should be kept apart here. The first is the experimental hit rate within a study, the proportion of synthesised candidates that showed the predicted activity, which several sources report and which is informative about that pipeline on that material. The second is the positive predictive value of a predictor, which requires that the tested candidates be representative of the predictions the model makes in its intended use; because candidates are typically selected by rank, and only the highest-ranked are tested, a hit rate measured on them is an upper bound rather than an estimate. Neither quantity is recoverable in aggregate from this literature, and the second would require a study design that none of the charted sources adopts.

### 4.5. Geographic Scope and the Limits of What Can Be Claimed

Charted by the botanical origin of the peptide material rather than by author affiliation, the evidence is concentrated in a relatively small number of named species, dominated by model organisms and major crops, with 9 of 79 charted sources specifying no botanical origin at all. No source identified within the scope of this search applied machine learning or deep learning to peptides from South African or other African plants.

That finding must be read against the design of the search, which materially limits what can be concluded. The strategy contained no African-specific geographic, botanical or ethnomedical terms; no regional or continental databases, such as African Journals Online or SciELO, were searched; no grey literature, theses or institutional repositories were consulted; and no preprint servers were searched. Research published in regional journals that are not indexed in Scopus, Web of Science, ScienceDirect or PubMed could not have been retrieved, irrespective of its quality. The absence reported here is therefore an absence within the indexed international literature as sampled by this search, and not evidence that no such work exists. A search designed around African sources, terms and indexes is a necessary prerequisite to any stronger claim, and this review does not make one.

The wider literature on natural-product drug discovery emphasises that biodiversity is unevenly represented in screening collections and that technological advances have renewed interest in underexplored taxa [94], and the review literature on peptides from medicinal plants makes the same point for peptide material specifically [43]. Reviews of plant-based natural-product discovery likewise describe ethnopharmacological knowledge as one of the established strategies for prioritising source material, alongside random screening and chemotaxonomic selection [95]. The present findings are consistent with these accounts while defining the gap more narrowly than it is usually stated: the specific combination of African plant material, peptide targets and machine learning methods was not identified here. That narrower formulation matters, because it identifies an opportunity to extend existing expertise in natural-product chemistry and ethnobotany into a computational domain, rather than implying an absence of capacity. That most of the predictors charted here are published and freely available is an opportunity rather than evidence that the practical barriers are low: reliable application also requires annotated sequence data for the relevant flora, computational resources, local methodological expertise and the capacity for experimental confirmation.

The more specific question the present findings raise is one of transferability. The predictors charted here were trained predominantly on sequences from model organisms and major crops, as Section 3.2.5 shows, and the single direct test of a general predictor on plant data in this evidence base returned a precision of 33.47% alongside an accuracy of 74.96% [25]. Whether models trained on well-characterised species retain their performance on taxa that are phylogenetically distant and poorly represented in the training data is therefore an open question, and one that bears directly on any attempt to apply these tools to African or other underrepresented flora. Answering it would require three things that the charted literature does not yet supply: annotated peptide sequence data from the flora in question, a held-out evaluation set drawn from those taxa rather than from the training distribution, and experimental confirmation of a sample of the resulting predictions. Until such an evaluation exists, applying existing predictors to underrepresented flora is a reasonable starting point but not a validated one.

### 4.6. Strengths and Limitations

The principal strength of this review is that its selection process is reproducible. Selection was executed from the retained database exports using a documented keyword filter whose complete term lists are published in Supplementary File S3 and whose output is listed record by record, and the filter was tested for recall before use. Every eligibility decision carries a criterion-level verdict with the supporting evidence quoted from the text, and every charted value is similarly evidenced. Any reader holding the four export files can reproduce the counts in Section 3.1 exactly. A second strength is the explicit operationalisation of three terms that this literature uses loosely: method as applied, validation and traditional-medicine linkage. Defining them is what makes the resulting counts interpretable.

Several limitations qualify the findings and are stated in order of the weight the authors attach to them.

First, the screening records generated at the protocol stage were incomplete, so selection was executed in full from the retained export files, and every count reported here derives from that process.

Second, the individual record-level decisions of the two reviewers were not retained in a form permitting comparison with one another or with the AI-assisted pass. No measure of inter-reviewer agreement can therefore be calculated, and none is reported. The available records also do not establish whether the two human reviewers assessed all 44 full texts retrieved separately during the AI-assisted pass. Prospective retention of record-level decisions would have removed this limitation at no cost.

Third, 60 records did not progress to full-text assessment because the available information was insufficient to establish eligibility, most commonly where no abstract was supplied. The 60 unresolved records are reported separately in Figure 1 and listed individually in Supplementary File S3 so that this source of possible selection bias remains visible. The review cannot establish whether any of them would have proved eligible had a full record been available.

Fourth, the PubMed search was amended during the review, as Section 2.4 describes, and both runs are reported. The union of two searches conducted on different dates is not equivalent to a single search conducted once, and records published between the two dates had unequal opportunity to be retrieved across the four databases.

Fifth, 80 sources met the eligibility criteria but only 79 are charted, the full text of the eightieth having proved unobtainable in a form permitting extraction. A further three reports were sought but not retrieved because of subscription paywalls and carry no eligibility decision. Four potentially eligible reports are therefore unaccounted for in the synthesis.

Sixth, no protocol was registered prospectively, and the eligibility criteria were modified during the conduct of the review, as Section 2.2 describes. The modifications are documented and their rationale stated, but documentation after the fact is a weaker safeguard than prospective registration.

Seventh, the search design limits the geographic claims, as set out in Section 4.5, and in particular cannot support any negative claim about the regional literatures that the four indexes do not cover.

Eighth, the charted counts for peptide class and species necessarily depend on the classification rules stated in Section 2.7 and Section 3.2.1. Those rules are published and the per-source assignments are listed, so a reader who prefers different boundaries can recompute; but the counts are not rule-independent, and sources addressing several classes are counted under each.

### 4.7. Implications for Research and Practice

Four implications follow from these findings, and they are ordered by how readily they can be acted upon.

First, reporting standards are the most pressing need and the most easily addressed. This review cannot rank computational methods because the evidence does not permit ranking: 45 of 61 empirical sources report no performance figure at all, and the 16 that do are not mutually comparable. The minimal four-item convention set out in Section 4.3 could be incorporated into publication standards, although some components, such as independent validation and same-dataset benchmarking, may require further data analysis or computation. Journals publishing in this area are well placed to require it.

Second, authors should state explicitly whether they are building a predictor or applying one. Both are legitimate and both are necessary, but they answer different questions and warrant different claims. A study that runs published tools over a new plant proteome contributes candidate sequences and biological insight; a study that trains and benchmarks a new model contributes methodological capability. Describing both as machine-learning-driven inflates the apparent methodological maturity of the field, as Section 3.2.2 quantifies, and deprives readers of information they need in order to use either result.

Third, the validation gap remains substantial, and the hydrolysate problem described in Section 4.4 should be named explicitly in reporting rather than left implicit. Where predicted sequences have been synthesised and assayed, that should be stated plainly; where only the parent hydrolysate or fraction was assayed, that should be stated equally plainly, and the predictions described as untested rather than validated. This costs nothing and materially improves the interpretability of the literature.

Fourth, documented ethnomedical knowledge is a substantially underused source of prior information for candidate selection. Overall, 8 sources in 79 make the link. Traditional-medicine systems encode sustained, systematic observation about which plant materials exert biological effects, which is precisely the form of prior that a prioritisation step is designed to exploit; ethnopharmacology-guided selection is an established strategy in natural-product discovery more broadly [94,95], and the small number of sources here that do make the link demonstrate that it is practicable for peptides specifically [16,43,53,55,58,83]. Extending plant-specific computational tools to South African and other African plant material, prioritised through documented ethnobotanical use, is an identified opportunity on the evidence assembled here, subject throughout to the search limitations described in Section 4.5.

## 5. Conclusions

This scoping review mapped the evidence on computational approaches to the discovery of plant-derived peptides, synthesising 79 charted sources identified through a documented and reproducible search of Scopus, Web of Science, ScienceDirect and PubMed. The charted literature includes 44 of 79 sources published in 2025 or the part of 2026 covered by the search, and it is broader than its antimicrobial origins: antioxidant and ACE-inhibitory peptides, drawn largely from the food science literature on protein hydrolysates, now account for a share of the evidence comparable to antimicrobial peptides.

It is also, methodologically, smaller than its own framing suggests. Overall, 11 of 79 charted sources trained and evaluated a machine learning model; the preponderance of the literature applies existing predictors, performs molecular docking or conducts conventional bioinformatic characterisation. Performance reporting is the field’s weakest point: roughly three-quarters of empirical sources report no performance figure, those that do report quantities that cannot be placed on a common scale, and only one empirical source in five benchmarks against a named comparator. No ranking of method families is supportable from this evidence, and this review deliberately offers none.

Three gaps are identified. Experimental confirmation of a source’s own computational prediction occurred in 18 of 62 applicable sources, leaving the majority of predictions untested by those who made them. Linkage between computational discovery and documented traditional-medicine knowledge occurred in 8 of 79 sources, leaving a large body of prior observation about plant bioactivity substantially unexploited. And no source applying machine learning to peptides from South African or other African plants was identified within the scope of this search, which was not designed to detect such work; this is therefore an absence within the indexed international literature sampled here rather than evidence of an absence of the work itself.

The methodological tools required to address these gaps already exist, are largely freely available and have been evaluated internationally. What the field lacks is not capability but convention. Future research should pair computational prediction with experimental confirmation of the named candidate sequences; report dataset composition, validation design, metric set and comparators to a common minimal standard; distinguish building a predictor from applying one; and record botanical provenance precisely, so that computational discovery can be connected to the botanical and ethnomedical knowledge that motivates it in the first place.

## Figures and Tables

**Table 1:** Population–Concept–Context framework used to define review eligibility.

Element	Description
Population	Plants as the source of the candidate peptide sequences. Any plant species qualifies, irrespective of medicinal reputation, economic importance or geographic origin. Studies in which a plant serves only as a heterologous expression host, and the peptide itself is not of plant origin, are excluded.
Concept	Application of any in silico approach (machine learning, deep learning, protein language modelling, conventional bioinformatics, genome or transcriptome mining, molecular docking or molecular dynamics simulation) to identify, predict, classify or design peptides. Method type is charted as an attribute of each source and is not used as an eligibility filter.
Context	Peer-reviewed journal articles, conference proceedings, narrative reviews, and descriptions of tools or databases, published in English between 2021 and 2026.

**Table 2:** Eligibility criteria applied at title and abstract screening and at full-text assessment.

Criterion	Inclusion	Exclusion
Peptide focus	Peptides are a subject of the work: bioactive peptides, meaning peptides with any measurable biological activity; antimicrobial peptides, the subset active against bacteria, fungi, viruses or parasites; or therapeutic peptides, those developed or intended for clinical use in humans	Peptides mentioned incidentally among other compound classes, or named only in passing
Plant source	The peptide originates from a plant; any species qualifies	Microbial, animal, marine, insect or wholly synthetic peptides; molecular pharming in which the plant is only an expression host
Computational component	Any in silico method applied by a primary study or by a tool or database description, or substantively discussed by a narrative review	No computational component, or a passing mention without substantive treatment
Publication type	Peer-reviewed primary study, narrative review, or description of a tool or database	Editorials, commentaries, conference abstracts without an accompanying full text, book chapters outside peer review
Language and date	English language, published 2021 to 2026	Languages other than English, or publication outside the date range

**Table 3:** Peptide classes addressed by the 79 charted sources. Sources addressing more than one class are counted under each, so the column sums to more than 79 and the percentages to more than 100%.

Peptide Class	*n*	% of 79
Antimicrobial, antibacterial or bacteriocin-type	33	41.8
Antioxidant	21	26.6
ACE-inhibitory or antihypertensive	18	22.8
Bioactive, functional class unspecified	18	22.8
Defensins	12	15.2
Antidiabetic, DPP-IV-inhibitory or glucose-lowering	11	13.9
Anticancer	9	11.4
Antifungal	8	10.1
Antiviral	8	10.1
Immunomodulatory	8	10.1
Cyclotides	8	10.1
Anti-inflammatory	7	8.9
Small secreted or small signalling peptides	7	8.9
Snakins and GASA-domain peptides	7	8.9
Lipid transfer proteins	5	6.3
sORF-, miRNA- or otherwise non-canonically encoded	4	5.1
Thionins	4	5.1
Neuroprotective	4	5.1
Cell-penetrating	2	2.5
Other named classes (RiPPs, cosmetic, anti-fatigue)	4	5.1

**Table 4:** Computational method as applied by each of the 79 charted sources. Each source is assigned to exactly one category, so the column sums to 79.

Method Category as Applied	*n*	% of 79	Model Trained and Evaluated by the Source
Bioinformatics prediction with molecular docking	23	29.1	No
None applied; methods discussed only	17	21.5	No
Previously published models applied in a pipeline	9	11.4	No; models taken from prior work
Conventional bioinformatics and gene-family analysis	7	8.9	No
Molecular docking and simulation only	7	8.9	No
Pretrained protein language model	5	6.3	Yes
Deep learning without a pretrained backbone	3	3.8	Yes
Deep learning applied to structure, not discovery	2	2.5	Yes, but not for discovery
Database or curated resource	2	2.5	No
Classical machine learning ensemble	2	2.5	Yes
Rule-based in silico simulation	1	1.3	No
Classical machine learning, single model	1	1.3	Yes
Total	79	100	11 of 79 (13.9%)

**Table 5:** Performance reporting across the 61 empirical sources. Per-source metric values, at the figures their sources give them and labelled with the data split on which each was measured, are reported in Supplementary File S4.

	*n*	%
Empirical sources (primary studies and tool or database descriptions)	61	100
Report at least one quantitative performance figure for a prediction task	14	23.0
Report structure-prediction confidence scores only	2	3.3
Report no performance figure	45	73.8
Sources that trained and evaluated a model of their own	11	18.0
of which report a prediction-task metric	10	n/a
of which report none	1	n/a
Sources applying existing tools without training a model	50	82.0
of which report a prediction-task metric	4	n/a
Sources benchmarking against at least one named computational comparator	13	21.3

## Data Availability

The keyword-filter terms, record-level screening decisions and data charting documentation are described in Supplementary Files S3 and S4. The original database exports were retained as the basis for the reported selection counts.

## Supplementary Materials

The following supporting information can be downloaded at: https://www.mdpi.com/article/10.3390/ijms27219639/s1.
