# Supplementary material for: Computational Discovery of Plant-Derived Peptides with Therapeutic Potential: A Scoping Review
Source: Int J Mol Sci. 2026 Oct 28;27(21):9639. doi: 10.3390/ijms27219639 (PMC13763164; doi:10.3390/ijms27219639)
Supplement: Supplementary file 1 [file ijms-27-09639-s001.zip › S1 Search_strategy.pdf]

Supplementary File S1. Search strategy

Computational Discovery of Plant-Derived Peptides with Therapeutic Potential: A Scoping Review

S1.1 Databases searched and yields

Four databases were searched. The exported files are the basis of every count reported in the manuscript and have been parsed directly for this revision. Counts were obtained by counting BibTeX entry headers in the Scopus file, TY records in each RIS file, and PMID records in the PubMed exports.

Database	Records in export	Export format	Date search run
Scopus	3,963	BibTeX (.bib)	17 August 2026
Web of Science	540	RIS (.ris)	17 August 2026
ScienceDirect	383 (four files of 100, 100, 100 and 83)	RIS (.ris)	17 August 2026
PubMed	440 (union of the two runs described in S1.4)	PubMed abstract format	17 August and 12 October 2026
Total identified	5,326	—	—

Two features of the export files affect how they are counted. The Scopus file is named "scopus 4007.bib" but contains 3,963 entries, and the file contents are the figure used throughout. The Web of Science file begins with a UTF-8 byte-order mark, so a line-anchored count of its records returns 539; the file contains 540.

S1.2 Date of searching

Scopus, Web of Science and ScienceDirect were searched on 17 August 2026. The export files carry no field recording the date of execution; the date is established from the authors' own browser history and is independently corroborated by the publication dates in the ScienceDirect export, in which 2026 records are distributed evenly from January to August at between 10 and 27 per month and then fall sharply from September onwards, to 9, 3, 3 and 9 records for September to December respectively. PubMed was searched on 17 August 2026 and again on 12 October 2026, following the amendment described in S1.5.

S1.3 Date restriction

All 4,886 records exported from Scopus, Web of Science and ScienceDirect carry a publication year between 2021 and 2026, and no record in any of those export files predates 2021. The date restriction was therefore applied at the point of searching, as a database-level limit, and not as a post hoc filter after retrieval.

S1.4 Search strings as run

Terms within each concept block were combined with OR and the blocks with AND. The fields searched were title, abstract and author keywords in each database. The full string was applied where database query-length limits permitted; the condensed string was substituted where they did not.

Full string as run (Scopus, Web of Science, ScienceDirect)

```
("artificial intelligence" OR "machine learning" OR "deep learning" OR "neural network*" OR "generative AI" OR "generative model*" OR "computational model*" OR "computational approach*" OR "bioinformatics" OR "in silico" OR "prediction model*" OR "predictive model*")

AND ("therapeutic peptide*" OR "bioactive peptide*" OR "antimicrobial peptide*" OR "plant antimicrobial peptide*" OR "plant-derived peptide*" OR "anticancer peptide*" OR "peptide drug*" OR "peptide therapeutic*" OR "cyclotide*" OR "peptide identification" OR "peptide prediction" OR "peptide design")

AND ("medicinal plant*" OR "plant-derived" OR "plant derived" OR "natural product*" OR "natural compound*" OR "phytochemical*" OR "plant species" OR "plant-based" OR "ethnobotan*" OR "plant bioactive compound*")

AND ("drug discovery" OR "drug development" OR "therapeutic discovery" OR "drug design" OR "peptide discovery" OR "peptide identification" OR "peptide prediction")
```

Condensed string as run (query-length-limited databases)

```
("artificial intelligence" OR "machine learning" OR "deep learning") AND ("therapeutic peptide" OR "bioactive peptide" OR "antimicrobial peptide") AND ("medicinal plant" OR "natural product" OR phytochemical)
```

S1.5 PubMed: the original search, its two faults, and the amended search

The PubMed search conducted on 17 August 2026 returned 30 records. Two independent faults in that search were identified during this revision.

First, every term in the string is enclosed in quotation marks and several carry a trailing asterisk. PubMed does not expand truncation inside a quoted phrase, so a term such as "neural network*" is handled as a literal phrase including the asterisk and matches almost nothing. Second, the date restriction was applied using the PubMed "last 5 years" filter rather than the fixed range of 2021 to 2026 applied to the other three databases. That filter is a rolling window measured backwards from the date of execution; applied on 17 August 2026 it covers 17 August 2021 onwards and excludes records published between 1 January and 16 August 2021.

The search was amended and run again on 12 October 2026 with both faults corrected: truncation is placed outside quoted phrases, MeSH headings are added where an appropriate heading exists, and the date limit is expressed as a fixed range matching the other databases. The corrected string returned 431 records.

Corrected PubMed string as run on 12 October 2026

```
("artificial intelligence"[tiab] OR "machine learning"[tiab] OR "deep learning"[tiab] OR neural network*[tiab] OR "computational biology"[Mesh] OR bioinformatic*[tiab] OR "in silico"[tiab] OR predictive model*[tiab])

AND (peptide*[tiab] OR "Peptides"[Mesh] OR "Antimicrobial Cationic Peptides"[Mesh] OR cyclotide*[tiab] OR defensin*[tiab])

AND ("Plants, Medicinal"[Mesh] OR "Plant Proteins"[Mesh] OR plant*[tiab] OR phyto*[tiab] OR medicinal plant*[tiab] OR "biological products"[Mesh] OR natural product*[tiab] OR ethnobotan*[tiab])

AND ("drug discovery"[tiab] OR "Drug Discovery"[Mesh] OR drug design[tiab] OR drug development[tiab] OR peptide discovery[tiab] OR peptide prediction[tiab])

AND 2021/01/01:2026/12/31[Date - Publication]
```

The corrected result set is not a superset of the original. Nine of the 30 records returned in August are absent from the 431 returned in October, because the two strings interrogate different field combinations. Both runs are therefore reported and their union, 440 unique records, is carried forward into the review. This is

recorded as a limitation in Section 4.6 of the manuscript: the union of two searches run on different dates is not equivalent to a single search run once.

S1.6 Known limitations of the strings as run

Three properties of these strings restrict what they could retrieve, and each bears directly on the coverage of the review.

First, the fourth concept block requires every retrieved record to describe itself in drug-discovery terms. A computational study of plant peptides need not do so, and several sources charted in this review would not satisfy that block. The conceptual framework of the revised manuscript does not retain this block, and a search without it would be expected to retrieve additional eligible records.

Second, truncation inside a quoted phrase is not expanded by all database engines, as described in S1.5.

Third, the strings contain no geographic, ethnobotanical, regional or continental terms, and no African journal platform, regional database, grey-literature source, thesis repository or preprint server was searched. Every statement in the manuscript about the geographic distribution of this literature is therefore a statement about the indexed international literature sampled by these strings, and not about the world literature.

S1.7 Deduplication and reproducibility

The four export files were merged and deduplicated in two sequential passes: first on digital object identifier, then on normalised title for records lacking an identifier or carrying an inconsistent one. Normalisation lower-cased the title and removed punctuation, diacritics and whitespace. This removed 264 duplicate records and left 5,062 unique records. Any reader holding the four export files can reproduce the figures of 5,326, 5,062 and 593 exactly.
