# Supplementary material for: Computational Discovery of Plant-Derived Peptides with Therapeutic Potential: A Scoping Review
Source: Int J Mol Sci. 2026 Oct 28;27(21):9639. doi: 10.3390/ijms27219639 (PMC13763164; doi:10.3390/ijms27219639)
Supplement: Supplementary file 1 [file ijms-27-09639-s001.zip › S2 PRISMA ScR checklist.pdf]

Supplementary File S2. Completed PRISMA-ScR checklist

Computational Discovery of Plant-Derived Peptides with Therapeutic Potential: A Scoping Review

The checklist follows Tricco, A.C.; Lillie, E.; Zarin, W.; et al. PRISMA Extension for Scoping Reviews (PRISMA-ScR): Checklist and Explanation. Ann. Intern. Med. 2018, 169, 467–473. Section and table numbers refer to the revised manuscript. PRISMA-ScR is the reporting guideline for scoping reviews; the flow of records is presented using the PRISMA 2020 flow diagram, which PRISMA-ScR adopts for this purpose.

Table S2.1. PRISMA-ScR checklist, with the location of each item in the revised manuscript.

#	Section	Item	Checklist item	Location	Status	How the item is reported
1	TITLE	Title	Identify the report as a scoping review.	Title	Yes	The title ends "A Scoping Review".
2	ABSTRACT	Structured summary	Provide a structured summary including, as applicable, background, objectives, eligibility criteria, sources of evidence, charting methods, results and conclusions that relate to the review questions and objectives.	Abstract	Yes	A single paragraph of 207 words covering each element. The journal guidance is about 200 words, so the abstract is at the upper limit; it carries no citations, as the journal requires.
3	INTRODUCTION	Rationale	Describe the rationale for the review in the context of what is already known. Explain why the review questions or objectives lend themselves to a scoping review approach.	Section 1	Yes	The first four paragraphs set out the two research traditions converging on plant peptides, the scale of the candidate space, the computational methods in use, and the four questions that remain unestablished. The fourth paragraph states that these concern the extent, composition and internal consistency of an evidence base, which is what a scoping review is designed to establish.
4	INTRODUCTION	Objectives	Provide an explicit statement of the questions and objectives being addressed with reference to their key elements or other relevant key elements used to conceptualise the review questions and objectives.	Section 1 (final paragraph); Section 2.2; Table 1	Yes	The final paragraph of Section 1 lists what is charted for every included source. Section 2.2 states the review question and two subsidiary questions, and Table 1 sets out the Population–Concept–Context framework.
5	METHODS	Protocol and registration	Indicate whether a review protocol exists; state if and where it can be accessed; and if available, provide registration information including the registration	Sections 2.10 and 4.6	Partially met	No protocol was registered prospectively in a public registry. Section 2.10 states this plainly and notes that the eligibility criteria were modified during the conduct of the review; the sixth limitation

			number.			in Section 4.6 repeats it. The two modifications and their rationale are recorded in Section 2.2.
6	METHODS	Eligibility criteria	Specify characteristics of the sources of evidence used as eligibility criteria (e.g., years considered, language, publication status), and provide a rationale.	Section 2.3; Table 2	Yes	Five criteria with inclusion and exclusion wording for each, covering peptide focus, plant source, computational component, publication type, and language and date. Two points of interpretation follow the table, including the asymmetry between primary studies and reviews on the computational criterion.
7	METHODS	Information sources	Describe all information sources in the search (e.g., databases with dates of coverage, contact with authors to identify additional sources) as well as the date the most recent search was executed.	Section 2.4; Supplementary File S1, Sections S1.1 and S1.2	Yes	Four databases with individual yields and the date of each search. The most recent search was executed on 12 October 2026. The amendment to the PubMed search and the reason for it are stated in Section 2.4 and reported in full in S1.5.
8	METHODS	Search	Present the full electronic search strategy for at least one database, including any limits used, such that it could be repeated.	Section 2.5; Supplementary File S1, Sections S1.4 and S1.5	Yes	Complete strings for all databases are reproduced verbatim, including both PubMed strings, the fields searched and the date limits. The known limitations of the strings as run are stated in S1.6.
9	METHODS	Selection of sources of evidence	State the process for selecting sources of evidence (i.e., screening and eligibility) included in the scoping review.	Sections 2.6 and 2.6.1; Supplementary File S3	Yes	Deduplication in two passes; the documented keyword filter and its recall test; criterion-level assessment of 593 records with the rule for assigning the determining criterion where more than one failed; retrieval of 91 full texts; and an explicit statement of what the two reviewers did and what the AI-assisted quality-assurance pass did.
10	METHODS	Data charting process	Describe the methods of charting data from the included sources of evidence (e.g., calibrated forms or forms that have been tested by the team before their use, and whether data charting was done independently or in duplicate) and any processes for obtaining and confirming data from investigators.	Section 2.7; Supplementary File S4	Yes	A 14-field form, piloted on ten sources and refined once. Charting was not performed in duplicate: each source was charted once from its full text, and every charted value was required to carry a verbatim quotation from that text as its evidential basis. Values for which no supporting quotation could be identified were recorded as not stated. Investigators were not contacted.
11	METHODS	Data items	List and define all variables for which data were sought and any assumptions and	Section 2.7	Yes	All 14 fields are listed. Four operational definitions are stated because they determine the reported counts: method as applied rather than as

			simplifications made.			discussed; validation as experimental confirmation by the source of its own prediction; traditional-medicine linkage as requiring a named system and a citation; and the rule for counting species in the provenance tally.
12	METHODS	Critical appraisal of individual sources of evidence	If done, provide a rationale for conducting a critical appraisal of included sources of evidence; describe the methods used and how this information was used in any data synthesis (if appropriate).	Section 2.9	Not applicable; justified	Formal critical appraisal was not undertaken, consistent with scoping review methodology. Section 2.9 states the rationale and describes what is charted in its place — reported performance, validation design, dataset composition and comparator benchmarking — so that a reader can judge the evidential strength of any individual source.
13	METHODS	Synthesis of results	Describe the methods of handling and summarising the data that were charted.	Section 2.8	Yes	Descriptive synthesis with frequencies and percentages. Denominators are named at each point of use (79 charted sources, 61 empirical sources, 67 sources for which the validation question arises). No meta-analysis was attempted and no ranking of method families is offered; the reason is given in Section 3.2.3.
14	RESULTS	Selection of sources of evidence	Give numbers of sources of evidence screened, assessed for eligibility and included in the review, with reasons for exclusions at each stage, ideally using a flow diagram.	Section 3.1; Figure 1	Yes	Every stage is reported with counts and reasons, and Figure 1 presents the full pathway as a PRISMA 2020 flow diagram: 5,326 identified, 264 duplicates removed, 5,062 screened, 4,469 removed by the keyword filter, 593 assessed at title and abstract, 442 excluded with six reasons, 60 unresolved, 91 reports sought, 3 not retrieved, 88 assessed at full text, 7 excluded with four reasons, 1 unresolved, 80 included and 79 charted. The 60 unresolved records and the 3 reports not retrieved are shown as distinct categories rather than absorbed into the exclusion counts.
15	RESULTS	Characteristics of sources of evidence	For each source of evidence, present characteristics for which data were charted and provide the citations.	Sections 3.2 to 3.2.7; Tables 3 to 5; Supplementary File S4	Yes	Characteristics are reported by category with supporting citations: study type, year of publication, peptide class (Table 3), method as applied (Table 4), performance reporting (Table 5), validation status, plant provenance, traditional-medicine linkage and geographic scope. Per-source values are listed in S4.

16	RESULTS	Critical appraisal within sources of evidence	If done, present data on critical appraisal of included sources of evidence (see item 12).	Section 2.9	Not applicable; justified	Not undertaken; see item 12.
17	RESULTS	Results of individual sources of evidence	For each included source of evidence, present the relevant data that were charted that relate to the review questions and objectives.	Supplementary File S4	Yes	All 14 charted fields are reported per source, including every performance metric at the value its source gives it, labelled with the data split on which it was measured, together with the verbatim quotation supporting each value.
18	RESULTS	Synthesis of results	Summarise and/or present the charting results as they relate to the review questions and objectives.	Sections 3.2.1 to 3.2.7	Yes	Synthesis by peptide class, computational method as applied, performance reporting, validation of the source's own predictions, plant provenance, traditional-medicine linkage and geographic scope of the plant material.
19	DISCUSSION	Summary of evidence	Summarise the main results (including an overview of concepts, themes and types of evidence available), link to the review questions and objectives, and consider the relevance to key groups.	Sections 4.1 to 4.5	Yes	Five numbered findings in Section 4.1, each tied to the charted data, followed by thematic discussion in Sections 4.2 to 4.5 in which each finding is interpreted, compared with the external literature, explained, and its implications and residual gaps identified.
20	DISCUSSION	Limitations	Discuss the limitations of the scoping review process.	Section 4.6	Yes	Eight limitations in order of the weight the authors attach to them: incomplete protocol-stage screening records; no retained reviewer-level decisions and therefore no agreement statistic; 60 unresolved records; the union of two PubMed runs conducted on different dates; four reports unaccounted for in the synthesis; no prospective registration; the geographic limits of the search; and the rule-dependence of the class and species counts.
21	DISCUSSION	Conclusions	Provide a general interpretation of the results with respect to the review questions and objectives, as well as potential implications and/or next steps.	Sections 4.7 and 5	Yes	Four implications ordered by how readily they can be acted upon, and a conclusion stating what the field lacks and what future research should report.
22	FUNDING	Funding	Describe sources of funding for the included sources of evidence, as well as sources of funding for the scoping review. Describe the role of the funders of the scoping	Back matter	Author action required	The Funding statement is to be completed by the authors before submission. Funding of the included sources was not charted and this is not reported.

			review.			

Summary of checklist status

Of the 22 checklist items, 18 are fully met, 2 are not applicable to a scoping review and the decision is justified in the manuscript, 1 is partially met, and 1 requires author action before submission.

Item 5, protocol and registration, is partially met: no protocol was registered prospectively, which is stated in Section 2.10 and reported as a limitation in Section 4.6. Item 22, funding, requires author action. Items 12 and 16 concern critical appraisal, which PRISMA-ScR treats as optional for scoping reviews; the decision not to undertake it is stated and justified in Section 2.9, and the information on which an appraisal would rest is charted and reported in its place.

Additional reporting

Two further matters are reported in the manuscript although the checklist does not require them. Section 2.6.1 describes the use of an AI-assisted tool for quality assurance, states that it did not act as a reviewer and did not constitute an independent second review, and identifies four features of the reviewer process that cannot be established from the available records and are therefore not reported. Section 2.6 states the rule by which a determining criterion was assigned where a record failed more than one criterion, and the per-record assignment is published in Supplementary File S3 so that the breakdown can be recomputed under a different convention.
