# Supplementary material for: Computational Discovery of Plant-Derived Peptides with Therapeutic Potential: A Scoping Review
Source: Int J Mol Sci. 2026 Oct 28;27(21):9639. doi: 10.3390/ijms27219639 (PMC13763164; doi:10.3390/ijms27219639)
Supplement: Supplementary file 1 [file ijms-27-09639-s001.zip › S3 Screening documentation.pdf]

Supplementary File S3. Screening documentation and record-level decisions

Computational Discovery of Plant-Derived Peptides with Therapeutic Potential: A Scoping Review

S3.1 Basis of the selection process

The screening records generated at the protocol stage were incomplete and their reported counts could not be reconciled with the retained record set. Rather than reproduce figures that could not be verified, the selection process was repeated in full from the four authentic database exports, in a form that any reader holding those exports can reproduce exactly. Every figure reported in Section 3.1 of the manuscript and in Figure 1 derives from the process documented here.

S3.2 The keyword filter

A documented keyword filter was applied to the 5,062 deduplicated records to define the subset read at title and abstract. A record entered that subset where its title, abstract or author keywords contained at least one peptide term and at least one plant term. Matching was case-insensitive and diacritic-insensitive, and records exported without an abstract were matched on title and author keywords alone. The filter retained 593 records and excluded 4,469.

The filter determined only which records were read. It made no eligibility decisions, and every record it retained was subsequently assessed against the five criteria in Table 2 of the manuscript.

Peptide terms (regular expression, case-insensitive)

```
peptid | cyclotide | defensin | thionin | snakin | lectin | \bAMPs?\b | \bPAMPs?\b | \bAFPs?\b |
lipid transfer protein | \bLTPs?\b | \bsORF | biopharmaceutic | protein hydrolysate
```

Plant terms (regular expression, case-insensitive)

```
\bplant | phyto | herbal | botanic | medicinal | natural product | ethnobotan | crop | \bflora\b |
arabidopsis | legume | cultivar | germplasm | agricultur | \bseed\b | \bleaf\b | \bleaves\b
```

S3.3 Recall test of the filter

A mechanical filter can in principle discard relevant literature without trace, so its recall was tested before use. All 24 studies included at the protocol stage that are present in the deduplicated pool were checked against the filter, and all 24 were retained, giving a recall of 24 of 24 on the only benchmark available. This does not establish that no eligible record was lost, since the benchmark is itself the product of the earlier search, but it does establish that the filter does not discard the literature the review was designed to find.

S3.4 How the determining criterion was assigned

Each of the 593 records was assessed against the five criteria in Table 2 of the manuscript, and a decision of include, exclude or uncertain was recorded with the determining criterion and a stated reason. Where a record failed more than one criterion, the determining criterion recorded is the lowest-numbered criterion

failed, in the order given in Table 2. Records for which the available text was insufficient to support a decision, principally those exported without an abstract, were recorded as uncertain rather than forced to a decision; they were not carried forward to retrieval and they were not excluded.

The per-record assignment is listed in Table S3.3, so that a reader applying a different convention for multiple failures can recompute the breakdown reported in Section 3.1 of the manuscript.

S3.5 Summary of decisions

Decision	n	Carried forward
Include — met all five criteria at title and abstract	91	Yes, to full-text retrieval
Exclude — failed at least one criterion	442	No
Uncertain — insufficient information to decide	60	No, and not excluded
Total read at title and abstract	593	—

Table S3.2. Exclusions at title and abstract by determining criterion.

Determining criterion	n
1. Peptide focus	109
2. Plant source	241
3. Computational component	88
4. Publication type	1
5. Language and date	1
Duplicate record not caught by the two deduplication passes	2
Total excluded	442

S3.6 Record-level decisions

Table S3.3 lists all 593 records read at title and abstract, with the database from which each was retrieved, the decision, the determining criterion where the decision was to exclude, and the recorded reason. Identifiers beginning with P denote records contributed by the PubMed search; the remainder are numbered sequentially within the merged Scopus, Web of Science and ScienceDirect pool.

Table S3.3. All 593 title and abstract decisions.

ID	Database	Decision	Determining criterion	Reason
0001	Scopus	Exclude	3. Computational component	No computational or in silico component
0002	Scopus	Exclude	3. Computational component	No computational or in silico component
0003	Scopus	Exclude	3. Computational component	No computational or in silico component
0004	Web of Science	Uncertain		
0005	Scopus	Exclude	3. Computational component	No computational or in silico component

0006	ScienceDirect	Exclude	1. Peptide focus	Not about peptides
0007	Scopus	Exclude	2. Plant source	Not plant-derived or plant-focused
0008	Scopus	Exclude	2. Plant source	Not plant-derived or plant-focused
0009	Scopus	Exclude	3. Computational component	No computational or in silico component
0010	Scopus	Exclude	2. Plant source	Not plant-derived or plant-focused
0011	Scopus	Exclude	2. Plant source	Not plant-derived or plant-focused
0012	Scopus	Exclude	1. Peptide focus	Not about peptides
0013	Scopus	Exclude	2. Plant source	Not plant-derived or plant-focused
0014	ScienceDirect	Exclude	3. Computational component	No computational or in silico component
0015	Scopus	Exclude	1. Peptide focus	Not about peptides
0016	Scopus	Exclude	3. Computational component	No computational or in silico component
0017	Scopus	Exclude	3. Computational component	No computational or in silico component
0018	Web of Science	Uncertain		
0019	Web of Science	Include		
0020	Scopus	Exclude	1. Peptide focus	Not about peptides
0021	Scopus	Exclude	1. Peptide focus	Not about peptides
0022	ScienceDirect	Exclude	2. Plant source	Not plant-derived or plant-focused
0023	Scopus	Exclude	1. Peptide focus	Not about peptides
0024	Scopus	Exclude	2. Plant source	Not plant-derived or plant-focused
0025	Scopus	Exclude	2. Plant source	Not plant-derived or plant-focused
0026	Scopus	Exclude	2. Plant source	Not plant-derived or plant-focused
0027	Scopus	Exclude	1. Peptide focus	Not about peptides
0028	ScienceDirect	Exclude	2. Plant source	Not plant-derived or plant-focused
0029	Web of Science	Include		
0030	Scopus	Exclude	3. Computational component	No computational or in silico component
0031	Scopus	Exclude	2. Plant source	Not plant-derived or plant-focused
0032	Scopus	Exclude	2. Plant source	Not plant-derived or plant-focused
0033	Scopus	Exclude	2. Plant source	Not plant-derived or plant-focused
0034	Scopus	Exclude	1. Peptide focus	Not about peptides
0035	Scopus	Exclude	3. Computational component	No computational or in silico component
0036	Scopus	Exclude	3. Computational component	No computational or in silico component
0037	Scopus	Exclude	3. Computational component	No computational or in silico component
0038	Scopus	Exclude	1. Peptide focus	Not about peptides
0039	Scopus	Exclude	2. Plant source	Not plant-derived or plant-focused
0040	Scopus	Exclude	2. Plant source	Not plant-derived or plant-focused

0041	Web of Science	Include		
0042	Scopus	Exclude	3. Computational component	No computational or in silico component
0043	Web of Science	Uncertain		
0044	Scopus	Include		
0045	Scopus	Exclude	3. Computational component	No computational or in silico component
0046	ScienceDirect	Exclude	1. Peptide focus	Not about peptides
0047	Web of Science	Uncertain		
0048	Web of Science	Include		
0049	Web of Science	Uncertain		
0050	Scopus	Include		
0051	Web of Science	Exclude	1. Peptide focus	Not about peptides
0052	ScienceDirect	Exclude	3. Computational component	No computational or in silico component
0053	Scopus	Exclude	2. Plant source	Not plant-derived or plant-focused
0054	Scopus	Exclude	2. Plant source	Not plant-derived or plant-focused
0055	Scopus	Exclude	1. Peptide focus	Not about peptides
0056	ScienceDirect	Exclude	1. Peptide focus	Not about peptides
0057	Scopus	Include		
0058	Scopus	Exclude	2. Plant source	Not plant-derived or plant-focused
0059	ScienceDirect	Exclude	2. Plant source	Not plant-derived or plant-focused
0060	Scopus	Exclude	2. Plant source	Not plant-derived or plant-focused
0061	Scopus	Exclude	1. Peptide focus	Not about peptides
0062	Scopus	Exclude	1. Peptide focus	Not about peptides
0063	Scopus	Exclude	2. Plant source	Not plant-derived or plant-focused
0064	Scopus	Exclude	2. Plant source	Not plant-derived or plant-focused
0065	Scopus	Uncertain		
0066	Scopus	Exclude	2. Plant source	Not plant-derived or plant-focused
0067	Scopus	Exclude	2. Plant source	Not plant-derived or plant-focused
0068	Scopus	Exclude	1. Peptide focus	Not about peptides
0069	ScienceDirect	Exclude	1. Peptide focus	Not about peptides
0070	Scopus	Include		
0071	Scopus	Exclude	2. Plant source	Not plant-derived or plant-focused
0072	Scopus	Exclude	2. Plant source	Not plant-derived or plant-focused
0073	Scopus	Exclude	1. Peptide focus	Not about peptides
0074	Scopus	Exclude	1. Peptide focus	Not about peptides
0075	Scopus	Exclude	2. Plant source	Not plant-derived or plant-focused
0076	Scopus	Exclude	2. Plant source	Not plant-derived or plant-focused
0077	Web of Science	Exclude	2. Plant source	Not plant-derived or plant-focused

0078	Scopus	Exclude	2. Plant source	Not plant-derived or plant-focused
0079	ScienceDirect	Include		
0080	Scopus	Exclude	2. Plant source	Not plant-derived or plant-focused
0081	Scopus	Exclude	2. Plant source	Not plant-derived or plant-focused
0082	Scopus	Exclude	3. Computational component	No computational or in silico component
0083	Scopus	Exclude	2. Plant source	Not plant-derived or plant-focused
0084	Scopus	Exclude	1. Peptide focus	Not about peptides
0085	Scopus	Exclude	2. Plant source	Not plant-derived or plant-focused
0086	Scopus	Exclude	2. Plant source	Not plant-derived or plant-focused
0087	Scopus	Exclude	2. Plant source	Not plant-derived or plant-focused
0088	Scopus	Exclude	2. Plant source	Not plant-derived or plant-focused
0089	Web of Science	Include		
0090	Scopus	Exclude	2. Plant source	Not plant-derived or plant-focused
0091	Scopus	Exclude	2. Plant source	Not plant-derived or plant-focused
0092	ScienceDirect	Exclude	1. Peptide focus	Not about peptides
0093	Scopus	Exclude	1. Peptide focus	Not about peptides
0094	ScienceDirect	Exclude	2. Plant source	Not plant-derived or plant-focused
0095	Scopus	Exclude	3. Computational component	No computational or in silico component
0096	Scopus	Exclude	3. Computational component	No computational or in silico component
0097	Scopus	Include		
0098	Scopus	Exclude	3. Computational component	No computational or in silico component
0099	Scopus	Exclude	2. Plant source	Not plant-derived or plant-focused
0100	Scopus	Exclude	2. Plant source	Not plant-derived or plant-focused
0101	Scopus	Include		
0102	Web of Science	Exclude	2. Plant source	Not plant-derived or plant-focused
0103	Scopus	Exclude	2. Plant source	Not plant-derived or plant-focused
0104	Scopus	Exclude	1. Peptide focus	Not about peptides
0105	Scopus	Include		
0106	ScienceDirect	Exclude	2. Plant source	Not plant-derived or plant-focused
0107	Scopus	Exclude	1. Peptide focus	Not about peptides
0108	Scopus	Exclude	2. Plant source	Not plant-derived or plant-focused
0109	Scopus	Exclude	2. Plant source	Not plant-derived or plant-focused
0110	Scopus	Exclude	2. Plant source	Not plant-derived or plant-focused
0111	Scopus	Uncertain		
0112	Scopus	Exclude	2. Plant source	Not plant-derived or plant-focused
0113	Scopus	Exclude	2. Plant source	Not plant-derived or plant-focused
0114	Scopus	Include		

0115	Scopus	Exclude	1. Peptide focus	Not about peptides
0116	Scopus	Exclude	2. Plant source	Not plant-derived or plant-focused
0117	Web of Science	Uncertain		
0118	Scopus	Exclude	1. Peptide focus	Not about peptides
0119	Scopus	Exclude	1. Peptide focus	Not about peptides
0120	Scopus	Exclude	3. Computational component	No computational or in silico component
0121	Scopus	Exclude	1. Peptide focus	Not about peptides
0122	Scopus	Exclude	1. Peptide focus	Not about peptides
0123	Scopus	Include		
0124	Scopus	Exclude	1. Peptide focus	Not about peptides
0125	Scopus	Uncertain		
0126	Scopus	Exclude	1. Peptide focus	Not about peptides
0127	Scopus	Exclude	3. Computational component	No computational or in silico component
0128	ScienceDirect	Exclude	3. Computational component	No computational or in silico component
0129	Scopus	Exclude	1. Peptide focus	Not about peptides
0130	ScienceDirect	Exclude	3. Computational component	No computational or in silico component
0131	Scopus	Exclude	2. Plant source	Not plant-derived or plant-focused
0132	Scopus	Include		
0133	Scopus	Uncertain		
0134	ScienceDirect	Exclude	1. Peptide focus	Not about peptides
0135	Scopus	Exclude	1. Peptide focus	Not about peptides
0136	Scopus	Exclude	3. Computational component	No computational or in silico component
0137	Scopus	Exclude	2. Plant source	Not plant-derived or plant-focused
0138	Scopus	Include		
0139	Scopus	Exclude	1. Peptide focus	Not about peptides
0140	Web of Science	Uncertain		
0141	Scopus	Exclude	2. Plant source	Not plant-derived or plant-focused
0142	Scopus	Uncertain		
0143	Scopus	Exclude	3. Computational component	No computational or in silico component
0144	Web of Science	Uncertain		
0145	Web of Science	Uncertain		
0146	Scopus	Exclude	3. Computational component	No computational or in silico component
0147	Scopus	Exclude	2. Plant source	Not plant-derived or plant-focused
0148	ScienceDirect	Exclude	2. Plant source	Not plant-derived or plant-focused

0149	Scopus	Exclude	1. Peptide focus	Not about peptides
0150	Scopus	Exclude	3. Computational component	No computational or in silico component
0151	Scopus	Exclude	2. Plant source	Not plant-derived or plant-focused
0152	Scopus	Exclude	1. Peptide focus	Not about peptides
0153	Scopus	Exclude	3. Computational component	No computational or in silico component
0154	Scopus	Exclude	1. Peptide focus	Not about peptides
0155	Scopus	Exclude	3. Computational component	No computational or in silico component
0156	Scopus	Exclude	1. Peptide focus	Not about peptides
0157	Scopus	Exclude	3. Computational component	No computational or in silico component
0158	Scopus	Exclude	2. Plant source	Not plant-derived or plant-focused
0159	ScienceDirect	Uncertain		
0160	Web of Science	Uncertain		
0161	Web of Science	Include		
0162	Scopus	Exclude	2. Plant source	Not plant-derived or plant-focused
0163	Scopus	Exclude	1. Peptide focus	Not about peptides
0164	ScienceDirect	Exclude	2. Plant source	Not plant-derived or plant-focused
0165	Web of Science	Uncertain		
0166	Scopus	Exclude	2. Plant source	Not plant-derived or plant-focused
0167	Scopus	Exclude	2. Plant source	Not plant-derived or plant-focused
0168	Scopus	Exclude	1. Peptide focus	Not about peptides
0169	Scopus	Exclude	1. Peptide focus	Not about peptides
0170	Scopus	Exclude	2. Plant source	Not plant-derived or plant-focused
0171	ScienceDirect	Exclude	2. Plant source	Not plant-derived or plant-focused
0172	Web of Science	Uncertain		
0173	Scopus	Exclude	3. Computational component	No computational or in silico component
0174	Scopus	Exclude	2. Plant source	Not plant-derived or plant-focused
0175	Scopus	Exclude	2. Plant source	Not plant-derived or plant-focused
0176	Scopus	Include		
0177	Scopus	Exclude	2. Plant source	Not plant-derived or plant-focused
0178	Scopus	Exclude	2. Plant source	Not plant-derived or plant-focused
0179	Scopus	Include		
0180	Web of Science	Uncertain		
0181	Scopus	Exclude	3. Computational component	No computational or in silico component
0182	ScienceDirect	Exclude	1. Peptide focus	Not about peptides
0183	Web of Science	Include		

0184	Scopus	Exclude	3. Computational component	No computational or in silico component
0185	Scopus	Exclude	2. Plant source	Not plant-derived or plant-focused
0186	Scopus	Include		
0187	Web of Science	Uncertain		
0188	Scopus	Exclude	2. Plant source	Not plant-derived or plant-focused
0189	Scopus	Exclude	3. Computational component	No computational or in silico component
0190	Web of Science	Uncertain		
0191	Scopus	Exclude	3. Computational component	No computational or in silico component
0192	Web of Science	Uncertain		
0193	Scopus	Exclude	1. Peptide focus	Not about peptides
0194	Scopus	Exclude	3. Computational component	No computational or in silico component
0195	Scopus	Include		
0196	Scopus	Exclude	2. Plant source	Not plant-derived or plant-focused
0197	ScienceDirect	Exclude	2. Plant source	Not plant-derived or plant-focused
0198	ScienceDirect	Include		
0199	Web of Science	Exclude	Duplicate record	Duplicate
0200	Scopus	Exclude	2. Plant source	Not plant-derived or plant-focused
0201	Scopus	Include		
0202	Scopus	Exclude	2. Plant source	Not plant-derived or plant-focused
0203	Scopus	Exclude	2. Plant source	Not plant-derived or plant-focused
0204	Scopus	Exclude	2. Plant source	Not plant-derived or plant-focused
0205	ScienceDirect	Exclude	2. Plant source	Not plant-derived or plant-focused
0206	Scopus	Uncertain		
0207	Scopus	Include		
0208	Scopus	Include		
0209	Scopus	Exclude	3. Computational component	No computational or in silico component
0210	Scopus	Exclude	1. Peptide focus	Not about peptides
0211	Scopus	Uncertain		
0212	Scopus	Uncertain		
0213	Scopus	Exclude	3. Computational component	No computational or in silico component
0214	Scopus	Exclude	3. Computational component	No computational or in silico component
0215	Scopus	Exclude	2. Plant source	Not plant-derived or plant-focused
0216	Scopus	Include		

0217	Web of Science	Include		
0218	Scopus	Exclude	2. Plant source	Not plant-derived or plant-focused
0219	Scopus	Exclude	2. Plant source	Not plant-derived or plant-focused
0220	Scopus	Exclude	2. Plant source	Not plant-derived or plant-focused
0221	Scopus	Exclude	1. Peptide focus	Not about peptides
0222	Scopus	Exclude	3. Computational component	No computational or in silico component
0223	Scopus	Exclude	2. Plant source	Not plant-derived or plant-focused
0224	Scopus	Exclude	2. Plant source	Not plant-derived or plant-focused
0225	Scopus	Exclude	1. Peptide focus	Not about peptides
0226	Web of Science	Include		
0227	Scopus	Include		
0228	Scopus	Exclude	2. Plant source	Not plant-derived or plant-focused
0229	Scopus	Exclude	2. Plant source	Not plant-derived or plant-focused
0230	Scopus	Include		
0231	Scopus	Include		
0232	ScienceDirect	Exclude	3. Computational component	No computational or in silico component
0233	Scopus	Exclude	2. Plant source	Not plant-derived or plant-focused
0234	Scopus	Exclude	3. Computational component	No computational or in silico component
0235	Web of Science	Include		
0236	Scopus	Include		
0237	Scopus	Exclude	2. Plant source	Not plant-derived or plant-focused
0238	Scopus	Exclude	2. Plant source	Not plant-derived or plant-focused
0239	Scopus	Exclude	2. Plant source	Not plant-derived or plant-focused
0240	Scopus	Exclude	3. Computational component	No computational or in silico component
0241	Scopus	Exclude	2. Plant source	Not plant-derived or plant-focused
0242	Scopus	Exclude	3. Computational component	No computational or in silico component
0243	Scopus	Exclude	2. Plant source	Not plant-derived or plant-focused
0244	Scopus	Exclude	1. Peptide focus	Not about peptides
0245	Scopus	Exclude	3. Computational component	No computational or in silico component
0246	ScienceDirect	Exclude	2. Plant source	Not plant-derived or plant-focused
0247	Scopus	Exclude	2. Plant source	Not plant-derived or plant-focused
0248	ScienceDirect	Include		
0249	Scopus	Exclude	2. Plant source	Not plant-derived or plant-focused
0250	Scopus	Exclude	2. Plant source	Not plant-derived or plant-focused
0251	Scopus	Include		

0252	ScienceDirect	Exclude	3. Computational component	No computational or in silico component
0253	Scopus	Exclude	3. Computational component	No computational or in silico component
0254	Scopus	Exclude	2. Plant source	Not plant-derived or plant-focused
0255	Web of Science	Exclude	2. Plant source	Not plant-derived or plant-focused
0256	Scopus	Exclude	2. Plant source	Not plant-derived or plant-focused
0257	Web of Science	Uncertain		
0258	Scopus	Exclude	1. Peptide focus	Not about peptides
0259	Web of Science	Uncertain		
0260	Scopus	Exclude	2. Plant source	Not plant-derived or plant-focused
0261	Scopus	Exclude	1. Peptide focus	Not about peptides
0262	Scopus	Exclude	2. Plant source	Not plant-derived or plant-focused
0263	Scopus	Exclude	2. Plant source	Not plant-derived or plant-focused
0264	Scopus	Exclude	2. Plant source	Not plant-derived or plant-focused
0265	Scopus	Exclude	2. Plant source	Not plant-derived or plant-focused
0266	Scopus	Exclude	1. Peptide focus	Not about peptides
0267	Scopus	Uncertain		
0268	Scopus	Exclude	3. Computational component	No computational or in silico component
0269	Scopus	Exclude	2. Plant source	Not plant-derived or plant-focused
0270	Web of Science	Exclude	2. Plant source	Not plant-derived or plant-focused
0271	Scopus	Exclude	3. Computational component	No computational or in silico component
0272	Scopus	Exclude	2. Plant source	Not plant-derived or plant-focused
0273	Scopus	Exclude	2. Plant source	Not plant-derived or plant-focused
0274	Scopus	Exclude	2. Plant source	Not plant-derived or plant-focused
0275	Scopus	Exclude	1. Peptide focus	Not about peptides
0276	Scopus	Exclude	1. Peptide focus	Not about peptides
0277	Scopus	Exclude	2. Plant source	Not plant-derived or plant-focused
0278	Scopus	Include		
0279	Scopus	Include		
0280	ScienceDirect	Exclude	3. Computational component	No computational or in silico component
0281	Web of Science	Include		
0282	ScienceDirect	Include		
0283	Scopus	Exclude	3. Computational component	No computational or in silico component
0284	Web of Science	Uncertain		
0285	Scopus	Exclude	2. Plant source	Not plant-derived or plant-focused
0286	Web of Science	Uncertain		

0287	Scopus	Exclude	3. Computational component	No computational or in silico component
0288	Scopus	Exclude	1. Peptide focus	Not about peptides
0289	Scopus	Uncertain		
0290	Scopus	Exclude	1. Peptide focus	Not about peptides
0291	Scopus	Exclude	2. Plant source	Not plant-derived or plant-focused
0292	Scopus	Uncertain		
0293	Scopus	Exclude	2. Plant source	Not plant-derived or plant-focused
0294	Web of Science	Uncertain		
0295	Scopus	Exclude	1. Peptide focus	Not about peptides
0296	ScienceDirect	Include		
0297	Scopus	Exclude	1. Peptide focus	Not about peptides
0298	Scopus	Exclude	2. Plant source	Not plant-derived or plant-focused
0299	Scopus	Exclude	1. Peptide focus	Not about peptides
0300	Scopus	Exclude	3. Computational component	No computational or in silico component
0301	Scopus	Include		
0302	Scopus	Exclude	4. Publication type	Ineligible publication type
0303	Scopus	Exclude	1. Peptide focus	Not about peptides
0304	Scopus	Exclude	3. Computational component	No computational or in silico component
0305	Scopus	Exclude	2. Plant source	Not plant-derived or plant-focused
0306	Scopus	Exclude	2. Plant source	Not plant-derived or plant-focused
0307	Scopus	Exclude	2. Plant source	Not plant-derived or plant-focused
0308	Scopus	Uncertain		
0309	Scopus	Exclude	2. Plant source	Not plant-derived or plant-focused
0310	ScienceDirect	Exclude	2. Plant source	Not plant-derived or plant-focused
0311	Scopus	Exclude	3. Computational component	No computational or in silico component
0312	Scopus	Exclude	2. Plant source	Not plant-derived or plant-focused
0313	Scopus	Exclude	3. Computational component	No computational or in silico component
0314	Scopus	Exclude	2. Plant source	Not plant-derived or plant-focused
0315	Scopus	Exclude	2. Plant source	Not plant-derived or plant-focused
0316	Web of Science	Include		
0317	Scopus	Exclude	3. Computational component	No computational or in silico component
0318	ScienceDirect	Exclude	2. Plant source	Not plant-derived or plant-focused
0319	Scopus	Exclude	Duplicate record	Duplicate
0320	Scopus	Exclude	2. Plant source	Not plant-derived or plant-focused
0321	Scopus	Exclude	3. Computational component	No computational or in silico component

0322	Scopus	Exclude	1. Peptide focus	Not about peptides
0323	Scopus	Exclude	2. Plant source	Not plant-derived or plant-focused
0324	Scopus	Exclude	2. Plant source	Not plant-derived or plant-focused
0325	Scopus	Uncertain		
0326	Scopus	Exclude	3. Computational component	No computational or in silico component
0327	Scopus	Exclude	2. Plant source	Not plant-derived or plant-focused
0328	Scopus	Uncertain		
0329	Scopus	Exclude	3. Computational component	No computational or in silico component
0330	Scopus	Exclude	1. Peptide focus	Not about peptides
0331	Scopus	Exclude	2. Plant source	Not plant-derived or plant-focused
0332	Scopus	Uncertain		
0333	Scopus	Include		
0334	Scopus	Exclude	3. Computational component	No computational or in silico component
0335	Scopus	Exclude	2. Plant source	Not plant-derived or plant-focused
0336	Scopus	Exclude	2. Plant source	Not plant-derived or plant-focused
0337	Scopus	Exclude	1. Peptide focus	Not about peptides
0338	Scopus	Exclude	3. Computational component	No computational or in silico component
0339	Scopus	Exclude	2. Plant source	Not plant-derived or plant-focused
0340	Scopus	Exclude	2. Plant source	Not plant-derived or plant-focused
0341	Web of Science	Include		
0342	Scopus	Exclude	2. Plant source	Not plant-derived or plant-focused
0343	Scopus	Exclude	2. Plant source	Not plant-derived or plant-focused
0344	Scopus	Exclude	3. Computational component	No computational or in silico component
0345	Scopus	Include		
0346	ScienceDirect	Exclude	2. Plant source	Not plant-derived or plant-focused
0347	Scopus	Exclude	2. Plant source	Not plant-derived or plant-focused
0348	Scopus	Exclude	2. Plant source	Not plant-derived or plant-focused
0349	Scopus	Exclude	2. Plant source	Not plant-derived or plant-focused
0350	Scopus	Exclude	3. Computational component	No computational or in silico component
0351	ScienceDirect	Include		
0352	Scopus	Exclude	3. Computational component	No computational or in silico component
0353	Scopus	Exclude	2. Plant source	Not plant-derived or plant-focused
0354	Scopus	Include		
0355	Scopus	Exclude	1. Peptide focus	Not about peptides
0356	Web of Science	Uncertain		

0357	Scopus	Uncertain		
0358	Scopus	Exclude	2. Plant source	Not plant-derived or plant-focused
0359	Scopus	Exclude	3. Computational component	No computational or in silico component
0360	Scopus	Exclude	2. Plant source	Not plant-derived or plant-focused
0361	Scopus	Uncertain		
0362	ScienceDirect	Exclude	2. Plant source	Not plant-derived or plant-focused
0363	Scopus	Exclude	3. Computational component	No computational or in silico component
0364	Web of Science	Exclude	2. Plant source	Not plant-derived or plant-focused
0365	Scopus	Include		
0366	Scopus	Uncertain		
0367	ScienceDirect	Exclude	2. Plant source	Not plant-derived or plant-focused
0368	ScienceDirect	Include		
0369	Scopus	Exclude	2. Plant source	Not plant-derived or plant-focused
0370	Scopus	Exclude	3. Computational component	No computational or in silico component
0371	Scopus	Exclude	1. Peptide focus	Not about peptides
0372	Scopus	Exclude	3. Computational component	No computational or in silico component
0373	Scopus	Exclude	2. Plant source	Not plant-derived or plant-focused
0374	Web of Science	Exclude	2. Plant source	Not plant-derived or plant-focused
0375	Scopus	Exclude	2. Plant source	Not plant-derived or plant-focused
0376	Scopus	Exclude	2. Plant source	Not plant-derived or plant-focused
0377	Scopus	Exclude	2. Plant source	Not plant-derived or plant-focused
0378	Scopus	Exclude	3. Computational component	No computational or in silico component
0379	Web of Science	Include		
0380	Scopus	Exclude	2. Plant source	Not plant-derived or plant-focused
0381	Scopus	Exclude	1. Peptide focus	Not about peptides
0382	Scopus	Exclude	2. Plant source	Not plant-derived or plant-focused
0383	Scopus	Exclude	2. Plant source	Not plant-derived or plant-focused
0384	Scopus	Exclude	2. Plant source	Not plant-derived or plant-focused
0385	Scopus	Exclude	2. Plant source	Not plant-derived or plant-focused
0386	Web of Science	Uncertain		
0387	Scopus	Exclude	2. Plant source	Not plant-derived or plant-focused
0388	Scopus	Exclude	3. Computational component	No computational or in silico component
0389	Scopus	Exclude	3. Computational component	No computational or in silico component
0390	Scopus	Include		
0391	Scopus	Exclude	2. Plant source	Not plant-derived or plant-focused

0392	Scopus	Exclude	2. Plant source	Not plant-derived or plant-focused
0393	Scopus	Exclude	2. Plant source	Not plant-derived or plant-focused
0394	Web of Science	Uncertain		
0395	Scopus	Exclude	2. Plant source	Not plant-derived or plant-focused
0396	ScienceDirect	Exclude	3. Computational component	No computational or in silico component
0397	Scopus	Include		
0398	ScienceDirect	Include		
0399	Scopus	Exclude	1. Peptide focus	Not about peptides
0400	Scopus	Exclude	2. Plant source	Not plant-derived or plant-focused
0401	Scopus	Exclude	2. Plant source	Not plant-derived or plant-focused
0402	ScienceDirect	Exclude	2. Plant source	Not plant-derived or plant-focused
0403	Scopus	Exclude	1. Peptide focus	Not about peptides
0404	Scopus	Exclude	2. Plant source	Not plant-derived or plant-focused
0405	Scopus	Exclude	2. Plant source	Not plant-derived or plant-focused
0406	Scopus	Exclude	2. Plant source	Not plant-derived or plant-focused
0407	Scopus	Exclude	2. Plant source	Not plant-derived or plant-focused
0408	Scopus	Exclude	2. Plant source	Not plant-derived or plant-focused
0409	Web of Science	Include		
0410	Web of Science	Include		
0411	Scopus	Exclude	1. Peptide focus	Not about peptides
0412	Scopus	Include		
0413	Scopus	Exclude	3. Computational component	No computational or in silico component
0414	Scopus	Exclude	2. Plant source	Not plant-derived or plant-focused
0415	Scopus	Uncertain		
0416	Web of Science	Include		
0417	Scopus	Exclude	2. Plant source	Not plant-derived or plant-focused
0418	Web of Science	Include		
0419	Scopus	Exclude	1. Peptide focus	Not about peptides
0420	Scopus	Exclude	3. Computational component	No computational or in silico component
0421	Scopus	Uncertain		
0422	Scopus	Exclude	1. Peptide focus	Not about peptides
0423	Scopus	Include		
0424	Web of Science	Include		
0425	Scopus	Include		
0426	Scopus	Exclude	3. Computational component	No computational or in silico component

0427	Scopus	Exclude	2. Plant source	Not plant-derived or plant-focused
0428	Scopus	Exclude	2. Plant source	Not plant-derived or plant-focused
0429	Scopus	Uncertain		
0430	Web of Science	Exclude	2. Plant source	Not plant-derived or plant-focused
0431	Scopus	Exclude	2. Plant source	Not plant-derived or plant-focused
0432	Web of Science	Include		
0433	Scopus	Exclude	2. Plant source	Not plant-derived or plant-focused
0434	Scopus	Exclude	2. Plant source	Not plant-derived or plant-focused
0435	Web of Science	Include		
0436	ScienceDirect	Exclude	2. Plant source	Not plant-derived or plant-focused
0437	Scopus	Exclude	1. Peptide focus	Not about peptides
0438	Scopus	Exclude	2. Plant source	Not plant-derived or plant-focused
0439	Scopus	Exclude	2. Plant source	Not plant-derived or plant-focused
0440	Scopus	Exclude	2. Plant source	Not plant-derived or plant-focused
0441	Scopus	Exclude	1. Peptide focus	Not about peptides
0442	Web of Science	Exclude	1. Peptide focus	Not about peptides
0443	Scopus	Exclude	1. Peptide focus	Not about peptides
0444	Scopus	Exclude	3. Computational component	No computational or in silico component
0445	Scopus	Exclude	3. Computational component	No computational or in silico component
0446	Scopus	Include		
0447	Scopus	Include		
0448	Scopus	Exclude	2. Plant source	Not plant-derived or plant-focused
0449	Scopus	Exclude	2. Plant source	Not plant-derived or plant-focused
0450	Scopus	Exclude	3. Computational component	No computational or in silico component
0451	Scopus	Exclude	2. Plant source	Not plant-derived or plant-focused
0452	Scopus	Exclude	2. Plant source	Not plant-derived or plant-focused
0453	Web of Science	Uncertain		
0454	Scopus	Exclude	1. Peptide focus	Not about peptides
0455	Web of Science	Exclude	2. Plant source	Not plant-derived or plant-focused
0456	Scopus	Exclude	2. Plant source	Not plant-derived or plant-focused
0457	Scopus	Exclude	2. Plant source	Not plant-derived or plant-focused
0458	Scopus	Exclude	3. Computational component	No computational or in silico component
0459	Web of Science	Include		
0460	Scopus	Exclude	3. Computational component	No computational or in silico component
0461	ScienceDirect	Exclude	2. Plant source	Not plant-derived or plant-focused
0462	Scopus	Exclude	2. Plant source	Not plant-derived or plant-focused

0463	Scopus	Exclude	1. Peptide focus	Not about peptides
0464	Web of Science	Exclude	1. Peptide focus	Not about peptides
0465	Scopus	Exclude	1. Peptide focus	Not about peptides
0466	Scopus	Exclude	2. Plant source	Not plant-derived or plant-focused
0467	Scopus	Exclude	2. Plant source	Not plant-derived or plant-focused
0468	Web of Science	Uncertain		
0469	Scopus	Include		
0470	Web of Science	Include		
0471	Scopus	Uncertain		
0472	Web of Science	Uncertain		
0473	Scopus	Exclude	1. Peptide focus	Not about peptides
0474	Scopus	Exclude	1. Peptide focus	Not about peptides
0475	Scopus	Exclude	2. Plant source	Not plant-derived or plant-focused
0476	Scopus	Exclude	2. Plant source	Not plant-derived or plant-focused
0477	Scopus	Exclude	2. Plant source	Not plant-derived or plant-focused
0478	Scopus	Exclude	2. Plant source	Not plant-derived or plant-focused
0479	Scopus	Uncertain		
0480	Scopus	Exclude	3. Computational component	No computational or in silico component
0481	Web of Science	Include		
0482	Scopus	Exclude	2. Plant source	Not plant-derived or plant-focused
0483	Scopus	Include		
0484	Scopus	Uncertain		
0485	Scopus	Exclude	2. Plant source	Not plant-derived or plant-focused
0486	Web of Science	Include		
0487	Scopus	Exclude	3. Computational component	No computational or in silico component
0488	ScienceDirect	Exclude	2. Plant source	Not plant-derived or plant-focused
0489	Web of Science	Exclude	2. Plant source	Not plant-derived or plant-focused
0490	Scopus	Exclude	2. Plant source	Not plant-derived or plant-focused
0491	Scopus	Exclude	2. Plant source	Not plant-derived or plant-focused
0492	Scopus	Exclude	2. Plant source	Not plant-derived or plant-focused
0493	Web of Science	Uncertain		
0494	Scopus	Exclude	2. Plant source	Not plant-derived or plant-focused
0495	Scopus	Uncertain		
0496	Web of Science	Include		
0497	ScienceDirect	Exclude	2. Plant source	Not plant-derived or plant-focused
0498	Scopus	Exclude	2. Plant source	Not plant-derived or plant-focused
0499	Scopus	Exclude	2. Plant source	Not plant-derived or plant-focused

0500	Scopus	Exclude	1. Peptide focus	Not about peptides
0501	Web of Science	Exclude	2. Plant source	Not plant-derived or plant-focused
0502	Scopus	Exclude	3. Computational component	No computational or in silico component
0503	Scopus	Include		
0504	Scopus	Exclude	3. Computational component	No computational or in silico component
0505	Web of Science	Uncertain		
0506	Scopus	Exclude	2. Plant source	Not plant-derived or plant-focused
0507	Scopus	Exclude	2. Plant source	Not plant-derived or plant-focused
0508	Scopus	Exclude	1. Peptide focus	Not about peptides
0509	Scopus	Exclude	2. Plant source	Not plant-derived or plant-focused
0510	Scopus	Exclude	2. Plant source	Not plant-derived or plant-focused
0511	Scopus	Exclude	2. Plant source	Not plant-derived or plant-focused
0512	Web of Science	Include		
0513	Scopus	Include		
0514	Scopus	Exclude	3. Computational component	No computational or in silico component
0515	Scopus	Exclude	2. Plant source	Not plant-derived or plant-focused
0516	Scopus	Exclude	2. Plant source	Not plant-derived or plant-focused
0517	Scopus	Exclude	2. Plant source	Not plant-derived or plant-focused
0518	Scopus	Include		
0519	Scopus	Uncertain		
0520	ScienceDirect	Exclude	2. Plant source	Not plant-derived or plant-focused
0521	Scopus	Exclude	2. Plant source	Not plant-derived or plant-focused
0522	Web of Science	Include		
0523	Scopus	Exclude	3. Computational component	No computational or in silico component
0524	Scopus	Exclude	2. Plant source	Not plant-derived or plant-focused
P045	PubMed	Exclude	1. Peptide focus	Excluded because peptides are not a subject of the work - the active components studied are flavonoids and 'peptide binding' is only an enrichment term - and no plant peptide is involved.
P046	PubMed	Exclude	2. Plant source	Excluded on C2: the peptidic natural products are microbial, from biosynthetic gene clusters of producer organisms, not plants.
P047	PubMed	Exclude	1. Peptide focus	Excluded because the subjects are synthetic small-molecule carbamate hybrids and the only peptide is the human amyloid-beta target, so neither C1 nor C2 holds.
P048	PubMed	Exclude	2. Plant source	Excluded on C2: this is genome mining of metagenomic bacterial biosynthetic gene clusters, so any peptides are microbial in origin.
P049	PubMed	Exclude	1. Peptide focus	Excluded because the compounds screened are plant small molecules and the peptide appears only in the name of the bacterial target enzyme, failing C1 and C2.
P050	PubMed	Exclude	1. Peptide focus	Excluded because the work surveys microbial BGCs of all classes with RiPPs only one among them, failing both the peptide-focus and plant-source criteria.

P051	PubMed	Exclude	1. Peptide focus	Excluded because this review covers marine actinobacterial secondary metabolites generally with peptides as one class among many, failing C1 and C2.
P052	PubMed	Exclude	1. Peptide focus	Excluded on C1 and C2: the study concerns flavonoid constituents of a herbal formula and no peptide is studied at all.
P053	PubMed	Exclude	2. Plant source	Excluded on C2: the lipopeptide originates from a marine cyanobacterium, not a plant.
P054	PubMed	Exclude	2. Plant source	Excluded on C2: the peptide sequences come from phage display libraries, not from a plant - only the conjugated iridoid small molecules are plant-derived.
P055	PubMed	Exclude	1. Peptide focus	Excluded because the screened compounds are microbial non-peptide natural products and the peptide element is only the protease target, failing C1 and C2.
P056	PubMed	Include		Included because bioactive peptides from soy protein hydrolysates are identified and characterised using applied molecular docking and dynamics in a 2025 English primary study.
P057	PubMed	Include		Included because DPP-IV inhibitory peptides derived from pumpkin seed protein are discovered via applied in silico screening and docking in a 2025 English primary study.
P058	PubMed	Uncertain		Uncertain because the plant source and computational work clearly qualify but it cannot be judged from the abstract whether cyclopeptide alkaloids count as bioactive peptides under C1 given triterpenoids are studied alongside them.
P059	PubMed	Include		Included because ACE inhibitory peptides from quinoa seed protein are predicted and validated using deep learning and docking in a 2025 English primary study.
P060	PubMed	Exclude	1. Peptide focus	Excluded on C1 and C2: the study is a metabolomics and network pharmacology analysis of a herbal decoction with no peptide subject.
old	PubMed	Exclude	1. Peptide focus	Comparative proteomics of plant extracellular vesicles; the subject is vesicle-associated proteins and contaminant discrimination, not bioactive, therapeutic or antimicrobial peptides.
P062	PubMed	Include		Included because plant-derived cyclotides are assessed as antiviral candidates using applied docking, MM-GBSA and MD in a 2026 English primary study.
P063	PubMed	Uncertain		Uncertain because this is a plant peptide database built with machine learning but the abstract does not establish that the uORF-encoded peptides are therapeutic, bioactive or antimicrobial rather than translational regulators.
P064	PubMed	Include		Included because bioactive peptides isolated from four medicinal plants are identified and docked against Alzheimer targets in a 2025 English primary study.
P065	PubMed	Exclude	1. Peptide focus	Excluded because the studied actives are plant polyphenols and the peptide reference is only the bacterial peptide deformylase target, failing C1 and C2.
P066	PubMed	Exclude	1. Peptide focus	Excluded because the subjects are synthetic thiazole compounds from a commercial library and the only peptide is the amyloid-beta target, failing C1 and C2.
P001	PubMed	Exclude	2. Plant source	Excluded because the peptides are microbial RiPPs from genome mining, so the plant-origin criterion fails.
P002	PubMed	Exclude	2. Plant source	Excluded because the glycopeptide antibiotics originate from Actinobacteria, not plants, so C2 fails.
P003	PubMed	Exclude	2. Plant source	Excluded because the tribrid natural product comes from microbial extracts rather than a plant, so C2 fails.
P004	PubMed	Exclude	2. Plant source	Excluded because the peptides are from a marine invertebrate and no computational method is applied, failing C2 and C3.

P005	PubMed	Uncertain		Uncertain because the database spans animal, plant and microbial toxins and the abstract does not show whether plant peptides are a substantive component.
P006	PubMed	Exclude	1. Peptide focus	Excluded because the work is about bacterial secondary metabolites generally, with microbial peptides only one class, failing C1 and C2.
P007	PubMed	Exclude	2. Plant source	Excluded because the lipopeptides are produced by a Bacillus strain, not a plant, so C2 fails.
old	PubMed	Exclude	3. Computational component	Full text read on mdpi.com. No dedicated section on computational peptide discovery and no named computational tool, algorithm or peptide database anywhere in the text; in silico work appears only as brief mentions in the Introduction and in four source-specific passages. Fails the computational cri
P009	PubMed	Exclude	2. Plant source	Excluded because the glycopeptide originates from a Streptomyces isolate rather than a plant, so C2 fails.
P010	PubMed	Include		Included because plant NCR peptides from Medicago truncatula were predicted with in silico AMP tools and tested, meeting all five criteria.
P011	PubMed	Exclude	2. Plant source	Excluded because the NRPs come from microbial and human microbiota genomes, not plants, so C2 fails.
P012	PubMed	Exclude	2. Plant source	Excluded because the adenylation domain predictions target microbial biosynthetic gene clusters, so the plant-source criterion fails.
P013	PubMed	Exclude	1. Peptide focus	Excluded because the review is dominated by small molecules with peptides incidental and no plant origin, failing C1 and C2.
old	PubMed	Exclude	2. Plant source	Antimicrobial peptides from Latin American biodiversity sourced from amphibians, reptiles, invertebrates and microorganisms; no plant-derived peptides are a subject of the work.
P015	PubMed	Exclude	2. Plant source	Excluded because the RiPP precursor peptides and maturases are of microbial origin, so C2 fails.
P016	PubMed	Exclude	2. Plant source	Excluded because the discovered NRP comes from Actinobacteria genomes rather than a plant, so C2 fails.
P017	PubMed	Exclude	1. Peptide focus	Excluded because peptides are one compound class among many and the sources are explicitly animal, marine and microbial, failing C1 and C2.
P018	PubMed	Exclude	2. Plant source	Excluded because the depsipeptides derive from marine-derived Acremonium fungi, not plants, so C2 fails.
P019	PubMed	Exclude	2. Plant source	Excluded because tetraselide comes from a marine-derived fungus rather than a plant, so C2 fails.
P020	PubMed	Exclude	1. Peptide focus	Excluded because peptides are one compound class among many in a synthetic-chemistry book chapter with no plant peptide source, failing C1, C2 and C4.
P021	PubMed	Include		Included because a maize-derived AMP was predicted with a computational AMP server and experimentally validated, meeting all five criteria.
P022	PubMed	Exclude	1. Peptide focus	Excluded because it benchmarks signal peptide prediction on bacterial phytoplasma genomes rather than bioactive plant peptides, failing C1 and C2.
P023	PubMed	Exclude	2. Plant source	Excluded because the peptides/proteins are fungal and oomycete pathogen effectors, not plant-derived bioactive peptides.
P024	PubMed	Exclude	1. Peptide focus	Excluded because the subjects are small-molecule phytochemicals, not peptides.
P025	PubMed	Exclude	1. Peptide focus	Excluded because peptides are only one incidental compound class among flavonoids, alkaloids, phenolics and terpenoids.
P026	PubMed	Exclude	1. Peptide focus	Excluded because the screened entities are small-molecule natural products from ZINC15 (coumarin and quinolizidine derivatives), not peptides.
P027	PubMed	Exclude	5. Language and date	Excluded on language: the bracketed title indicates a Chinese-language article, so the English-language criterion fails.

P028	PubMed	Exclude	2. Plant source	Excluded because the peptides are microbial nonribosomal products, not plant-derived, and the piece reads as a short commentary.
P029	PubMed	Exclude	1. Peptide focus	Excluded because peptides are one passing class within a broad phytochemical docking review.
old	PubMed	Exclude	2. Plant source	Cyclic peptides discovered by mRNA display from synthetic and genetically encoded libraries against the human GIP receptor; not plant-derived. The item is a Chemical Science highlight commenting on another paper rather than a primary study or substantive review.
P031	PubMed	Exclude	2. Plant source	Excluded because the peptides are bacterial RiPPs (graspetides and spliceotides) from environmental microbial genomes, not plant-derived.
P032	PubMed	Exclude	1. Peptide focus	Excluded because it mines bacterial biosynthetic gene clusters for secondary metabolites generally, failing both the peptide-focus and plant-source criteria.
P033	PubMed	Exclude	1. Peptide focus	Excluded because the subject is snake venom enzymes and their mostly non-peptide inhibitors, not plant-derived peptides.
P034	PubMed	Exclude	1. Peptide focus	Excluded because the bioactive agent is a plant triterpenoid and the peptides are only proteomic identification products.
P035	PubMed	Exclude	1. Peptide focus	Excluded because the actives are plant small molecules and the only peptides are epitopes designed from a bacterial protein.
P036	PubMed	Exclude	2. Plant source	Excluded because the peptides are microbial nonribosomal peptides, not plant-derived.
P037	PubMed	Exclude	1. Peptide focus	Excluded because it concerns structure prediction of food proteins generally, with no focus on bioactive or therapeutic peptides.
P038	PubMed	Exclude	1. Peptide focus	Excluded because the study explicitly seeks non-peptide inhibitors, and its libraries are marine as well as herbal.
P039	PubMed	Exclude	2. Plant source	Excluded because the peptides are bacterial and fungal nonribosomal products, not of plant origin.
P040	PubMed	Uncertain		Uncertain because the record has no title and only a one-sentence abstract fragment, which is not enough to judge any criterion, although the phrase non-peptidic is suggestive of a peptide-focus failure.
P041	PubMed	Include		Included because in silico AMP prediction was applied to discover and characterise antimicrobial peptides from the plant Amaranthus tricolor.
P042	PubMed	Exclude	2. Plant source	Excluded because the peptide studied is from parasitoid wasp venom, with plant knottins only a comparison set.
P043	PubMed	Include		Included because it is a deep-learning framework built and benchmarked specifically for plant peptide-protein interaction prediction.
P044	PubMed	Exclude	1. Peptide focus	Excluded because the peptides are food-authentication markers rather than bioactive peptides, no in silico method is applied, and the paper is from 2020.
old	PubMed	Include		Full text read on academic.oup.com. Antimicrobial peptides are a substantive subject, with their own section, Table 1 of 12 AMP databases and Table 2 comparing tools, and a dedicated subsection on AMP databases of plant sources describing PhytAMP, PlantAFP and PlantPepDB, three plant-specific resour
old	PubMed	Include		Full text read on frontiersin.org. Cyclotides from Clitoria ternatea, described by the authors as a medicinal plant. Applied: FRODOCK 2.0 rigid-body protein-peptide docking with in-house PPCheck scoring; Maestro and Protein Preparation Wizard with PROPKA and OPLS3e; Desmond System Builder with TIP4P
old	PubMed	Include		Passed title and abstract screening. Full text could not be obtained: the DOI redirects to linkinghub.elsevier.com and sciencedirect.com refuses automated retrieval. No eligibility decision taken. Reported in the reports-not-retrieved box of the flow diagram.
